# Preclinical evaluation of DOTAGA.Glu.(FAPI)_2_ and DO3A.Glu.(FAPI)_2_ as theranostics with human dosimetry extrapolation to lutetium-177 and terbium-161 analogs

**DOI:** 10.1007/s00259-025-07565-9

**Published:** 2025-10-17

**Authors:** Adrianna Bilinska, Naveen Kumar, Silvano Gnesin, Tilman Läppchen, Elena Menéndez, Marcel Martin, Frank Rösch, Axel Rominger, Eleni Gourni

**Affiliations:** 1 Department of Nuclear Medicine, Inselspital, Bern University Hospital, Bern, Switzerland; 2 Graduate School of Cellular and Biomedical Sciences, University of Bern, Bern, Switzerland; 3 Institute of Radiation Physics, Lausanne University Hospital and University of Lausanne, Lausanne, Switzerland; 4 Department of Chemistry–TRIGA site, Johannes Gutenberg-University Mainz, Mainz, Germany

**Keywords:** FAP, Dimeric FAP inhibitors, Gallium-68, Lutetium-177, Terbium-161, Dosimetry

## Abstract

**Purpose:**

This study aims to assess DOTAGA.Glu.(FAPI)_2_ and DO3A.Glu.(FAPI)_2_, specifically engineered as precursors for the development of theranostic FAPI-targeted radioligands.

**Methods:**

DOTAGA.Glu.(FAPI)_2_ and DO3A.Glu.(FAPI)_2_ were radiolabeled with gallium-68 and lutetium-177, followed by in vitro (lipophilicity, protein binding, saturation, internalization and externalization) studies on FAP^+^ CAFs. In vivo (biodistribution, metabolic stability, blood kinetics, PET/SPECT/CT imaging) and ex vivo, (autoradiography, immunohistochemistry) conducted on PC3-mice. Murine dosimetry data were extrapolated to human estimates.

**Results:**

All radioligands achievied > 98% radiochemical purity, demonstrating high FAP affinity (K_d_:0.7–0.9 nM) and rapid internalization in CAFs, with differences in lipophilicity and serum protein binding. In vivo studies, for [^68^Ga]Ga-DOTAGA.Glu.(FAPI)_2_ and [^68^Ga]Ga-DO3A.Glu.(FAPI)_2_ showed high and sustained tumor uptake up to 3 h p.i. (18–19%I.A./g). For [^177^Lu]Lu-DOTAGA.Glu.(FAPI)_2_ and [^177^Lu]Lu-DO3A.Glu.(FAPI)_2_ tumor uptake was 16.2 ± 2.5 and 15 ± 1.2% IA/g at 4 h p.i., reaching 5.1 ± 0.1 and 2.8 ± 0.4%IA/g at 48 h, respectively. All radioligands exhibited low blood retention levels. PET/SPECT/CT imaging confirmed high tumor-to-background ratios. Uptake patterns correlate well with autoradiography images of heterogeneous FAP distribution in PC3-mice, while the detection of both murine and human FAP in PC3-tumors was demonstrated through immunohistochemistry. The extrapolated human absorbed dose estimates (Gy/GBq) for [^177^Lu]Lu-DOTAGA.Glu.(FAPI)_2_ were generally higher across most organs compared to [^177^Lu]Lu-DO3A.Glu.(FAPI)_2_. Human extrapolation of the ^161^Tb-labeled radioligands delivered on average ~ 38% higher absorbed doses in tissues as compared to their ^177^Lu-labeled counterparts.

**Conclusion:**

These results support the potential clinical translation of DOTAGA.Glu.(FAPI)_2_ and DO3A.Glu.(FAPI)_2_, as promising candidates for precise diagnosis and treatment of FAP-expressing malignancies.

**Supplementary Information:**

The online version contains supplementary material available at 10.1007/s00259-025-07565-9.

## Introduction

Nuclear Medicine is increasingly focusing on radiolabeled FAP inhibitors (FAPIs) for cancer management. These radioligands target fibroblast activation protein (FAP), showing strong potential in radiotheranostics. FAP is a cell-surface serine protease mainly found on cancer-associated fibroblasts (CAFs), with generally low expression in healthy tissues. Its overexpression in cancers allows for selective radiopharmaceutical delivery to the tumor microenvironment with minimal effect on healthy tissues [1, 2].

FAPI-radiopharmaceuticals derived from UAMC1110 [3] have shown promise preclinically and clinically [4–25]. However, early FAPI-monomers wash-out quickly from the tumor lesions, limiting their therapeutic effect [11, 26]. Newer FAPIs aim to improve tumor retention by modifying precursor chemistry [9, 15, 17, 18, 24] and using short-lived radionuclides like yttrium-90 [9].

To enhance tumor residency, researchers developed homodimeric FAPIs [10, 12, 15, 21, 22, 24]. An early example is DOTAGA.(SA.FAPI)_2_ by Moon et al. [18], labeled with gallium-68 and lutetium-177 and evaluated in U87MG- and PC3-mice [12, 18]. Clinically, [^177^Lu]Lu-DOTAGA.(SA.FAPI)_2_ was investigated in radioiodine-refractory differentiated thyroid cancer (RR-DTC) and other malignancies showing tumor retention up to one week, with the colon considered the dose-limiting organ [6, 26]. [^177^Lu]Lu-DOTAGA.(SA.FAPI)_2_ compared to its monomeric version, [^177^Lu]Lu-DOTA.SA.FAPI [26], delivered higher tumor doses, however, a need to improve its pharmacokinetic performance was essential. To address this, Martin et al. introduced a second-generation of dimers, DO3A.Glu.(FAPI)_2_ and DOTAGA.Glu.(FAPI)_2_ [15], where a central glutamic acid (Glu) linker connects the FAPIs and the chelator. Replacing the squaramide linker (SA) with glutamic acid (Glu) was a strategic choice to enhance hydrophilicity, leading to faster clearance from non-target tissues and improved performance.

Following Martin et al.’s synthesis and the initial preclinical evaluation [15], the present study provides a comprehensive assessment of DO3A.Glu.(FAPI)_2_ and DOTAGA.Glu.(FAPI)_2_ labeled with gallium-68 and lutetium-177. All radioligands were evaluated in vitro (lipophilicity, protein binding, saturation, internalization and externalization studies) on immortalized FAP^+^ CAFs. In vivo (biodistribution, PET/SPECT/CT imaging, metabolic stability, blood kinetics) and ex vivo (autoradiography and immunohistochemistry) studies along with dosimetry were performed using PC3-mice. Furthermore, murine dosimetry data were extrapolated to human estimates for [^177^Lu]Lu-DOTAGA.Glu.(FAPI)_2_, [^177^Lu]Lu-DO3A.Glu.(FAPI)_2_, [^161^Tb]Tb-DOTAGA.Glu.(FAPI)_2_ and [^161^Tb]Tb-DO3A.Glu.(FAPI)_2_.

## Materials and methods

### Radiolabeling/quality control/lipophilicity/protein binding studies

DOTAGA.Glu.(FAPI)_2_ and DO3A.Glu.(FAPI)_2_ were radiolabeled with gallium-68 (20 nmol each), and with lutetium-177 (5 nmol each). Their radiochemical purity and stability were assessed by radio- RP-HPLC and TLC over 4 h for the ^68^Ga- and 96 h for the ^177^Lu-labeled compounds. The lipophilicity (LogD_octanol/PBS_) and protein binding were determined as described in the supplemental data.

### Cell lines

The human prostate adenocarcinoma cell line PC3 and the prostate-derived CAF cells were used in the present study. Cultivation conditions, materials and further details are described in the supplemental data.

### Saturation binding studies/internalization/externalization

For receptor saturation studies, FAP^+^ CAFs were incubated with 0.1 to 10 nM of the radioligands. For internalization studies, approximately 2.5 pmol of the radioligands were added to CAFs followed by incubation for 15, 30, 60, 90, 120, 180 and 240 min for the ^68^Ga-labeled or 30, 60, 120, 240 and 360 min for the ^177^Lu-labeled radioligands at 37 °C, 5% CO_2_. For the externalization studies, approximately 2.5 pmol of the ^177^Lu-labeled radioligands were added to CAFs followed by incubation for 2 h at 37 °C, 5% CO_2_. The amount of externalized activity was measured at 0, 10, 20, 30, 60, 120, 240, and 1440 min (Supplemental Data).

### Animal models

Male athymic Balb/C nude mice (6 weeks/20–25 g) were implanted with PC3 cells (3.5 × 10^6^/100 µL PBS) into their right shoulder to develop tumors. The animals were used for biodistribution and PET/SPECT/CT imaging studies, once tumors reached 250–300 mm^3^.

### Biodistribution studies

600 pmol (~ 4 MBq/100 µL NaCl 0.9%) of [^68^Ga]Ga-DOTAGA.Glu.(FAPI)_2_ and [^68^Ga]Ga-DO3A.Glu.(FAPI)_2_ were intravenously injected into PC3-mice and biodistribution studies were conducted at 1, 2, and 3 h p.i. 600 pmol (~ 4 MBq/100 µL NaCl 0.9%) of [^177^Lu]Lu-DOTAGA.Glu.(FAPI)_2_ and 450 pmol (~ 2.6 MBq/100 µL NaCl 0.9%) of [^177^Lu]Lu-DO3A.Glu.(FAPI)_2_ were injected intravenously into PC3-mice, with biodistribution at 4, 24, 48, 72 and 96 h p.i.

To demonstrate the specificity of binding, the same as above amount of each radioligand co-injected with 20 nmol of unlabeled precursor (100 µL total), with biodistribution at 1 h and 4 h p.i. for the the ^68^Ga- and the ^177^Lu-labeled radioligands, respectively.

All mice were euthanized with intraperitoneal injection of pentobarbital (150 mg/kg), and selected organs were dissected, weighed, and measured for radioactivity using a gamma-counter. Results are expressed as % injected activity per gram tissue (% I.A./g), presented as mean ± SD (*n* = 3–4).

### Metabolic stability

Approximately 1 mL of blood from PC3-mice was collected to heparinized tubes at 30 min p.i. of 600 pmol of [^177^Lu]Lu-DOTAGA.Glu.(FAPI)_2_ and 450 pmol of [^177^Lu]Lu-DO3A.Glu.(FAPI)_2_ (*n* = 3). Following blood collection, plasma was injected into RP-HPLC (Supplemental Data).

### Blood and organs clearance kinetics

Healthy mice (*n* = 2) were intravenously injected with 600 pmol (~ 4 MBq/100 µL NaCl 0.9%) of [^177^Lu]Lu-DOTAGA.Glu.(FAPI)_2_ and 450 pmol (~ 2.6 MBq/100 µL NaCl 0.9%) of [^177^Lu]Lu-DO3A.Glu.(FAPI)_2_. Blood samples were collected from the facial vein at 1, 3, 5, 7, 10, 15, 20, 30, 45, 60, 240, and 1440 min p.i., weighed, and measured for radioactivity using a γ-counter. Results were expressed as % I.A./g of blood and plotted over time. The initial and terminal half-lives were determined using Two-Phase Decay Model. The biological half-lives of selected organs were calculated from the biodistribution data using One-Phase Decay Model (GraphPad Prism 8).

### Small-animal PET/SPECT/CT imaging

PET/CT scans were acquired using a dedicated micro-PET/SPECT/CT scanner. Static PET images were obtained at 1, 2 and 3 h after injection of 600 pmol (~ 4 MBq/100 µL) of [^68^Ga]Ga**-**DOTAGA.Glu.(FAPI)_2_ and [^68^Ga]Ga**-**DO3A.Glu.(FAPI)_2_ in PC3-mice (*n* = 3/group) (Supplemental Data).

### Ex vivo autoradiography

Frozen tumor Sect. (7 μm) from PC3-mice (*n* = 3) injected with 600 pmol (~ 13 MBq/100 µL) of [^177^Lu]Lu-DOTAGA.Glu.(FAPI)_2_ and 450 pmol (~ 11 MBq/100 µL) of [^177^Lu]Lu-DO3A.Glu.(FAPI)_2_ were exposed to a Autoradiography Cassette FBCS 810 (FisherBiotech) overnight. Cassettes were scanned using a Cyclone Plus analyzer (PerkinElmer). The images were analyzed with OptiQuant software.

### Immunohistochemistry

Immunohistochemical staining was conducted using frozen tumor Sect. (7 μm) from the same tumors used for autoradiography, with consecutive sections taken directly from the autoradiography samples (Supplemental Data).

The stained sections were analyzed using a Zeiss LSM980 Confocal Microscop and Zen 3.4 Software.

### Dosimetry analysis

Biodistribution kinetics for [^177^Lu]Lu-DOTAGA.Glu.(FAPI)_2_ and [^177^Lu]Lu-DO3A.Glu.(FAPI)_2_ were assessed from the biodistribution data at 4, 24, 48, 72, and 96 h p.i. (*n* = 3). The %I.A./g of tissue was calculated for liver, lungs, stomach, spleen, pancreas, kidneys, intestines, bone, muscle, salivary glands, heart wall, blood, tumor. To estimate the absolute activity in each source organ, the individual %I.A./g values were multiplied by the corresponding organ mass derived from the standard 25 g MOBY mouse phantom [27], yielding in the normalized source organ activities (nA) per time point and animal.

Following Cicone et al. [28], the nA values for source organs were corrected to account for the fraction of administered activity sequestered by the tumor, as described in more detail in the dosimetry section of the supplemental data. According to the MIRD formalism [29], time-integrated activity coefficients (TIACs) were derived by integrating the normalized time-activity curves (nTACs), which were fitted using the bi-exponential functions. TIACs were calculated by fitting the nTACs with the bi-exponential functions and performing time integration in MATLAB. Additionally, mass-normalized TIACs (TIAC/g) were derived using organ masses from the standard 25 g MOBY mouse phantom (Supplemental data).

### Extrapolation of murine TIACs to human equivalents

The TIACs, for [^177^Lu]Lu-DOTAGA.Glu.(FAPI)_2_ and [^177^Lu]Lu-DO3A.Glu.(FAPI)_2_, were determined by time integration of the nTACs. To address inter-animal variability, the TIACs for each source organ were calculated from both the mean nTACs and the nTACs obtainend from fitting the mean ± 1 standard deviation (SD) of the normalized time-activity data, providing upper and lower bounds.

The mice-to-human TIAC extrapolation accounted for species-specific differences in relative source organ masses, m(organ), as compared to the total body weight (WB), for the mice (m) and human (h) models respectively [28, 30–32].

The human organ masses, m(organ)_h_, were obtained from ICRP-89 adult male and female reference phantoms [33], while the murine organ masses, m(organ)_m_, were derived from the standard MOBY mouse model [27]. The extrapolated human TIACs, TIAC_h_ were entered in the OLINDA/EXM software [34] to compute the absorbed doses to target organs and the effective dose for adult male, female, and gender-averaged human models.

Human dosimetry for the same compounds was estimated assuming radiolabeling with terbium-161 instead of lutetium-177. Given the comparable physical half-lives (6.95 days for terbium-161, 6.7 days for lutetium-177) and assuming identical biological distribution, we approximated TIAC_h, Tb−166_ =TIAC_h, Lu−177_. The decay data for terbium-161 were substituted for those of lutetium-177 in OLINDA calculations, while all other parameters remained unchanged.

### Statistical analysis

A two-way ANOVA was used to assess differences in biodistribution between each pair of the [⁶⁸Ga]- and [¹⁷⁷Lu]-labelled radiopharmaceuticals across organs and timepoints. Analyses were performed using GraphPad Prism 8, with statistical significance set at *P* < 0.05 (95% confidence level).

## Results

### Radiolabeling/quality control of the radioligands/stability

**Fig. 1:**
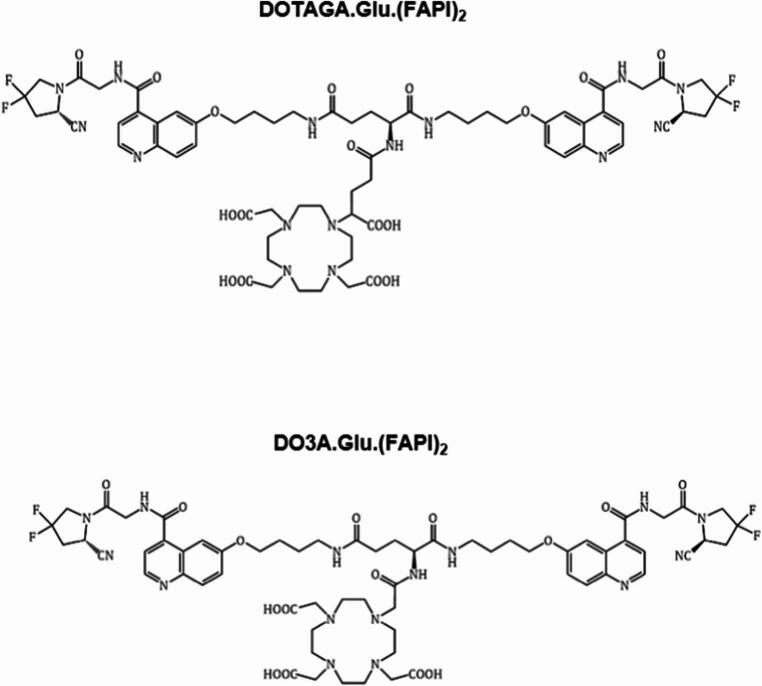
Schematic representations of DOTAGA.Glu.(FAPI)_2_ and DO3A.Glu.(FAPI)_2_

DOTAGA.Glu.(FAPI)_2_ and DO3A.Glu.(FAPI)_2_ (Fig. 1) were labelled with gallium-68 at > 98% radiochemical purity, with apparent molar activity (A_m_) of 8–14 GBq/µmol (not decay corrected). For the ^177^Lu-labeled radioligands, A_m_ were 6–38 GBq/µmol, depending on the study. No colloid formation was observed.

The formulated ^68^Ga-labeled and ^177^Lu-labeled FAPI radioligands remained highly stable, with no radiolysis or chemical decomposition observed up to 4 and 96 h post-labeling, respectively. (Supplementary data, Fig. S2-S6).

### Lipophilicity/protein binding studies

The LogD_octanol/PBS−pH7.4_ data and the % to human serum proteins binding are depicted in Table 1. The LogD_octanol/PBS_ values indicated a more hydrophilic nature of [^68^Ga]Ga- and [^177^Lu]Lu-DOTAGA.Glu.(FAPI)_2_ (−2.9 and − 3, respectively) compared to [^68^Ga]Ga- and [^177^Lu]Lu-DO3A.Glu.(FAPI)_2_ (−2.2 and − 1.7, respectively).

**Table 1:** LogD_octanol/PBS−pH7.4_, % human serum protein bound activity at 30 and 60 min of incubation and HPLC retention times

Radioligand	LogD	% Protein binding (30 min)	% Protein binding (60 min)	Retention time (minutes)
[ ^68^ Ga]Ga-DOTAGA.Glu.(FAPI) _2_ [ ^177^ Lu]Lu-DOTAGA.Glu.(FAPI) _2_ [ ^68^ Ga]Ga-DO3A.Glu.(FAPI) _2_ [ ^177^ Lu]Lu-DO3A.Glu.(FAPI) _2_	−2.9 ± 0.1 −3.0 ± 0.1 −2.2 ± 0.04 −1.7 ± 0.01	9.4 ± 2.2 9.2 ± 0.03 9.6 ± 0.2 27.7 ± 0.9	6.3 ± 0.4 7.4 ± 0.5 6.3 ± 0.3 28.3 ± 1.8	11’47 11’36 11’51 11’45

Both [^68^Ga]Ga-DOTAGA.Glu.(FAPI)_2_ and [^68^Ga]Ga-DO3A.Glu.(FAPI)_2_ demonstrated similar binding to human serum proteins after 30 and 60 min. [^177^Lu]Lu-DO3A.Glu.(FAPI)_2_ exhibited approximately three times higher protein binding compared to [^177^Lu]Lu-DOTAGA.Glu.(FAPI)_2_.

### Saturation binding/internalization/externalization studies

**Fig. 2:**
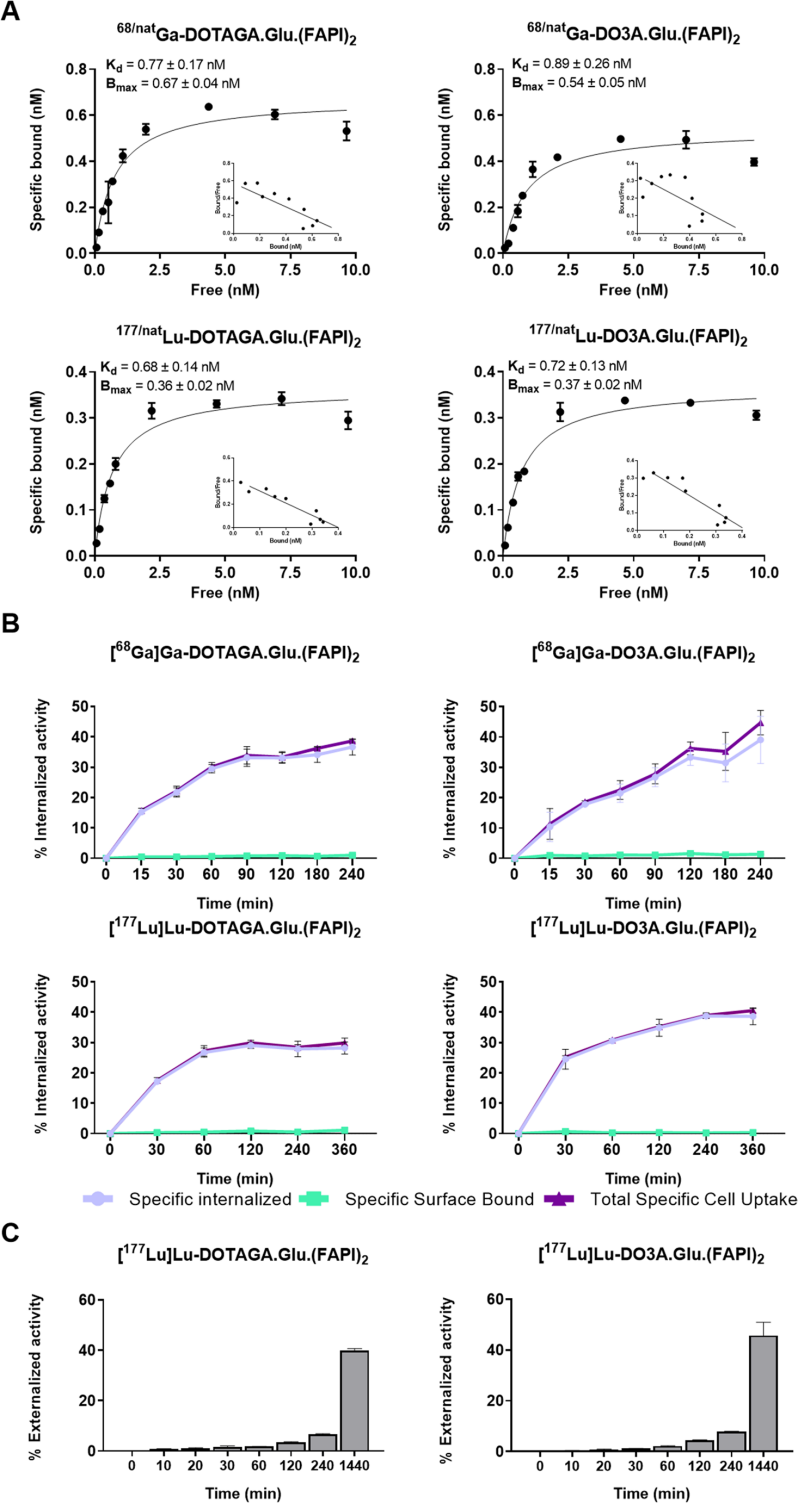
**(A)** Saturation binding on CAFs, using 0.1 to 10 nM of ^68/nat^Ga-DOTAGA.Glu.(FAPI)_2_, ^68/nat^Ga-DO3A.Glu.(FAPI)_2_, ^177/nat^Lu-DOTAGA.Glu.(FAPI)_2_ and ^177/nat^Lu-DO3A.Glu.(FAPI)_2_; K_d_) and B_max_ were calculated via nonlinear regression (GraphPad Prism 8). **(B)** Internalization and surface binding assessed in CAFs at 37 °C with [^68^Ga]Ga-DOTAGA.Glu.(FAPI)_2_, [^68^Ga]Ga-DO3A.Glu.(FAPI)_2_, [^177^Lu]Lu-DOTAGA.Glu.(FAPI)_2_ and [^177^Lu]Lu-DO3A.Glu.(FAPI)_2_. Total specific cell uptake calculated as specific surface bound fraction plus specific internalized fraction (% of total applied radioactivity). Nonspecific binding was determined in the presence of 1 µM UAMC1110. **(C)** Externalized activity expressed as % of total internalized activity (100%)

^68/nat^Ga-DOTAGA.Glu.(FAPI)_2_, ^68/nat^Ga-DO3A.Glu.(FAPI)_2_, ^177/nat^Lu-DOTAGA.Glu.(FAPI)_2_ and ^177/nat^Lu-DO3A.Glu.(FAPI)_2_ exhibited similar affinities for FAP^+^ CAFs, with K_d_ values of 0.8 ± 0.2 nM, 0.9 ± 0.3 nM, 0.7 ± 0.1 nM and 0.7 ± 0.1 nM respectively (Fig. 2A). They showed similar B_max_ values (0.7 ± 0.04 nM, 0.5 ± 0.05 nM, 0.4 ± 0.02 nM and 0.4 ± 0.02 nM, respectively), corresponding to ~ 3.6 × 10^5^ receptors per cell.

All the radioligands rapidly internalized into CAFs, with over 30% of the total added activity internalized within 60 min at 37 °C (Fig. 2B).

[^177^Lu]Lu-DOTAGA.Glu.(FAPI)_2_ and [^177^Lu]Lu-DO3A.Glu.(FAPI)_2_ showed similar retention in CAFs. After 24 h at 37 °C, ~ 40% and ~ 46% of the initially internalized activity was released [^177^Lu]Lu-DOTAGA.Glu.(FAPI)_2_ and [^177^Lu]Lu-DO3A.Glu.(FAPI)_2_, respectively (Fig. 2C).

Blocking studies using 1 µM UAMC1110 confirmed the high specificity to FAP^+^ CAFs showing negligible nonspecific binding.

### Biodistribution studies of [^68^Ga]Ga-DOTAGA.Glu.(FAPI)_2_ and [^68^Ga]Ga-DO3A.Glu.(FAPI)_2_

**Fig. 3:**
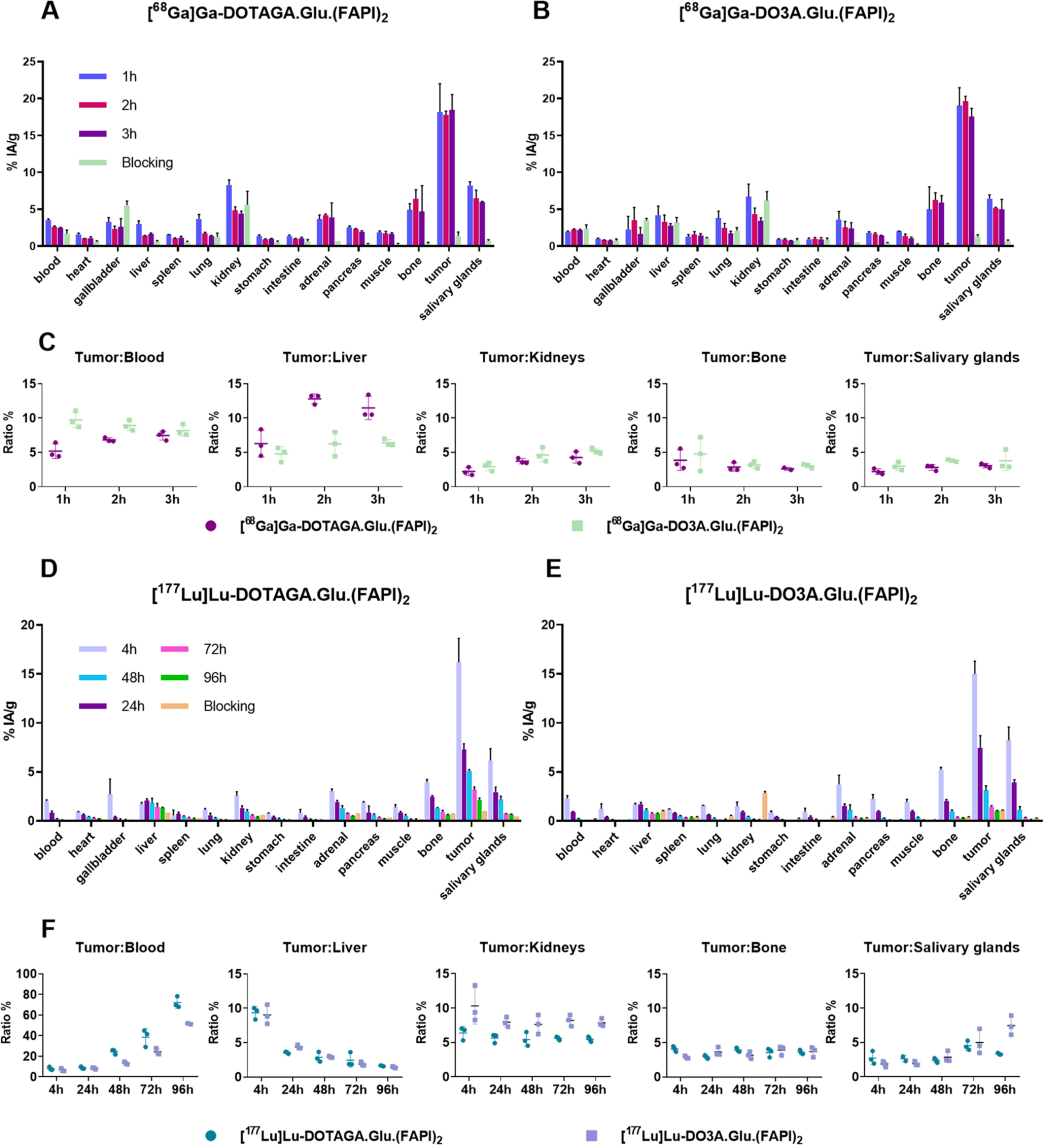
Biodistribution data of **(A)** [^68^Ga]Ga-DOTAGA.Glu.(FAPI)_2_ and **(B)** [^68^Ga]Ga-DO3A.Glu.(FAPI)_2_ in PC3-mice at 1, 2, and 3 h p.i including blocking studies at 1 h p.i. (**C**) Tumor-to-background ratios at the same time points. Biodistribution data of **(D)** [^177^Lu]Lu-DOTAGA.Glu.(FAPI)_2_ and (**E**) [^177^Lu]Lu-DO3A.Glu.(FAPI)_2_ at 4, 24, 48, 72 and 96 h p.i. with blocking at 4 h p.i. in PC3-mice. (F) Tumor-to-background ratios at same time points.All data have been calculated as % I.A./g of tissue and are presented as mean±SD(n=3)

Biodistribution data for [^68^Ga]Ga-DOTAGA.Glu.(FAPI)_2_ and [^68^Ga]Ga-DO3A.Glu.(FAPI)_2_ and their tumor-to-background ratios in PC3-mice are presented in Fig. 3A-C and Table S1. [^68^Ga]Ga-DOTAGA.Glu.(FAPI)_2_ and [^68^Ga]Ga-DO3A.Glu.(FAPI)_2_ showed high and consistent tumor uptake at all three time points. [^68^Ga]Ga-DOTAGA.Glu.(FAPI)_2_ exhibited uptake of 18.2 ± 2.3, 17.8 ± 0.5 and 18.4 ± 2.2%I.A./g at 1, 2 and 3 h p.i., respectively. [^68^Ga]Ga-DO3A.Glu.(FAPI)_2_ demonstrated comparable uptake of 19.0 ± 2.4, 19.6 ± 0.7 and 17.5 ± 1.1%I.A./g at the same time points. Blood pool retention was low for both: [^68^Ga]Ga-DOTAGA.Glu.(FAPI)_2_ declined from 3.5 ± 0.2%I.A./g to 2.5 ± 0.1%I.A./g over 3 h, and [^68^Ga]Ga-DO3A.Glu.(FAPI)_2_ remained stable around 2%I.A./g. Liver uptake was slightly higher for [^68^Ga]Ga-DO3A.Glu.(FAPI)_2_ at all three time points. Kidney uptake patterns were similar for both, while increased uptake in bones and salivary glands likely reflects endogenous FAP expression. [^68^Ga]Ga-DOTAGA.Glu.(FAPI)_2_ compared to [^68^Ga]Ga-DO3A.Glu.(FAPI)_2_ showed elevated tumor-to-liver values at all time points, indicating more favorable pharmacokinetics.

### Biodistribution studies of [^177^Lu]Lu-DOTAGA.Glu.(FAPI)_2_ and [^177^Lu]Lu-DO3A.Glu.(FAPI)_2_

Our previous study on the role of circulating FAP in the pharmacokinetics of FAPI-based radiopharmaceuticals, including DOTAGA.Glu.(FAPI)_2_ and DO3A.Glu.(FAPI)_2_, emphasized the importance of the precise mass dosing for optimal in vivo performance [35]. Based on these findings, and combined with our current in vitro data showing higher lipophilicity of [^68^Ga]Ga-DO3A.Glu.(FAPI)_2_ and [^177^Lu]Lu-DO3A.Glu.(FAPI)_2_ versus their DOTAGA.Glu.(FAPI)_2_ counterparts, we refined the dosing: reducing [^177^Lu]Lu-DO3A.Glu.(FAPI)_2_ from 600 to 450 pmol, while maintaining 600 pmol for [^177^Lu]Lu-DOTAGA.Glu.(FAPI)_2_, to enhance their tumor-to-liver ratio and overall efficacy.

Biodistribution data and their tumor-to-background ratios of in PC3-mice are shown in Fig. 3D-F and Tables S2-3. [^177^Lu]Lu-DOTAGA.Glu.(FAPI)_2_ and [^177^Lu]Lu-DO3A.Glu.(FAPI)_2_ showed similar organ distribution to their ^68^Ga-labelled analogs, with ~ 15%I.A./g tumor uptake at 4 h p.i., dropping by ~ 50% at 24 h. At 48 h, [^177^Lu]Lu-DOTAGA.Glu.(FAPI)_2_ had significantly higher tumor uptake (5.1 ± 0.1%I.A./g) than [^177^Lu]Lu-DO3A.Glu.(FAPI)_2_ (3.1 ± 0.5%I.A./g; *p* < 0.0001), and remained higher even at 96 h (2.2 ± 0.2 vs. 1.0 ± 0.03%I.A./g; *p* < 0.001). Both showed low background and blood activity (~ 2%I.A./g) at 4 h. Elevated uptake in salivary glands and bone was observed. Clearance from tumor, salivary glands, and bone was similar, however [^177^Lu]Lu-DOTAGA.Glu.(FAPI)_2_ revealed higher tumor-to-blood ratios, indicating better pharmacokinetics.

### Metabolic stability

**Fig. 4:**
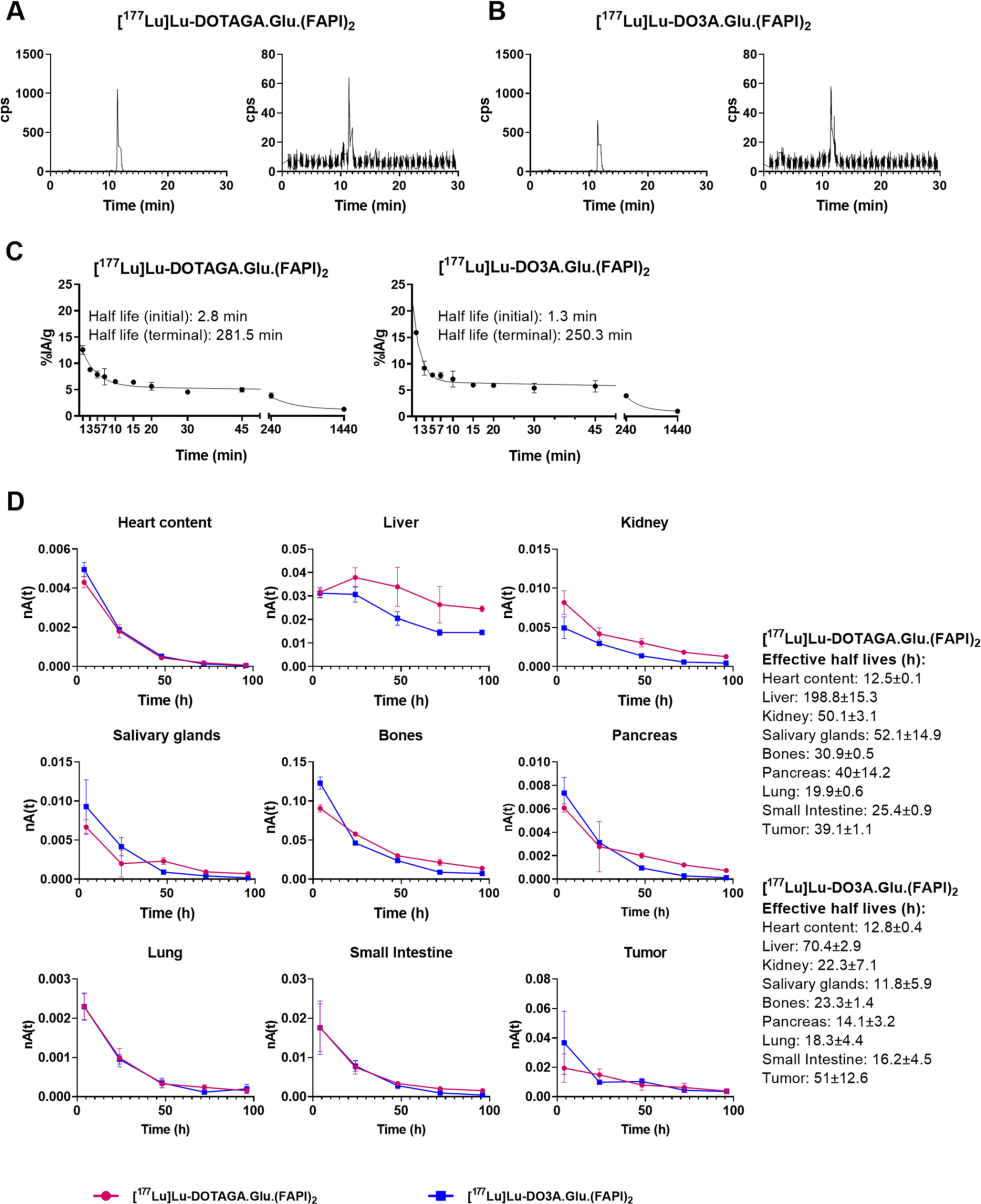
Representative radio-HPLC chromatograms of **(A)** [^177^Lu]Lu-DOTAGA.Glu.(FAPI)_2_ and **(B)** [^177^Lu]Lu-DO3A.Glu.(FAPI)_2_ before injection (left) and from blood samples at 30 min p.i. (right). **(C)** Blood clearance over time (*n* = 2) and **(D)** Normalized time–activity curves (nTACs) for [^177^Lu]Lu-DOTAGA.Glu.(FAPI)_2_ and [^177^Lu]Lu-DO3A.Glu.(FAPI)_2_ corrected for tumor “sink” effects (*n* = 3). Liver effective half-life from the mono-exponential fit exceeded 160 h and was limited at lutetium-177’s physical half-life

The radio-HPLC analysis of mouse plasma samples at 30 min p.i. showed that the circulating radioactivity consisted exclusively of intact ^177^Lu-labeled radioligands in both cases (Fig. 4A-B).

### Blood and organs clearance kinetics

Figure 4C shows the blood clearance curves of [^177^Lu]Lu-DOTAGA.Glu.(FAPI)_2_ and [^177^Lu]Lu-DO3A.Glu.(FAPI)_2_. The initial and terminal half-lives were slightly longer for [^177^Lu]Lu-DOTAGA.Glu.(FAPI)_2_ (2.8 and 282 min) than for [^177^Lu]Lu-DO3A.Glu.(FAPI)_2_ (1.3 and 250 min). The organ clearance curves and their biological half-lives are shown in Fig. S6 and Table S4. [^177^Lu]Lu-DOTAGA.Glu.(FAPI)_2_ exhibited prolonged tissue retention, including PC3-tumors.

### Small-animal PET/SPECT/CT imaging

**Fig. 5:**
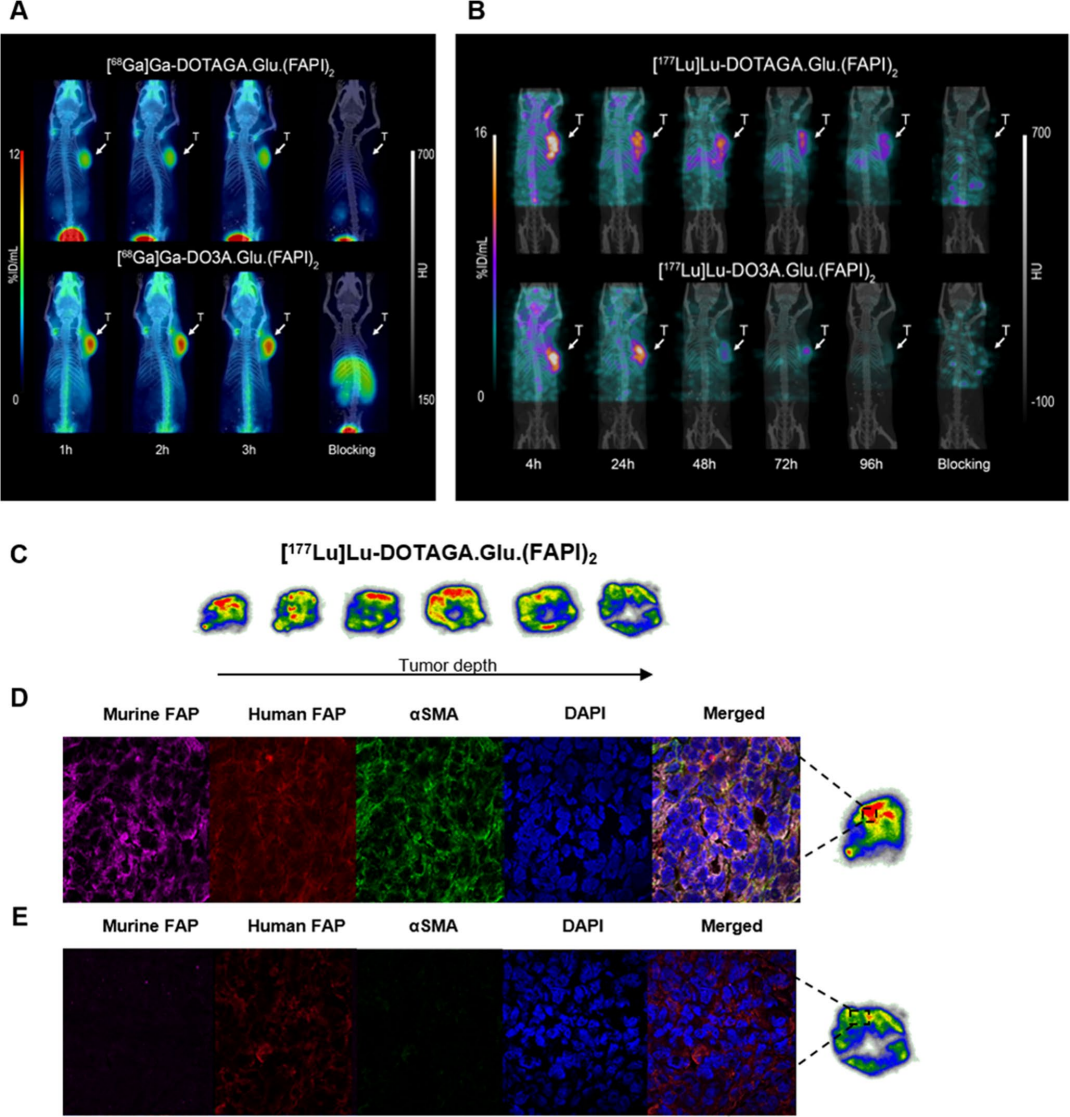
**(A)** PET/CT images of [^68^Ga]Ga-DOTAGA.Glu.(FAPI)_2_ and [^68^Ga]Ga-DO3A.Glu.(FAPI)_2_ in PC3-mice at 1, 2, and 3 h p.i. with blocking at 1 h p.i. **(B)** SPECT/CT images of [^177^Lu]Lu-DOTAGA.Glu.(FAPI)_2_ and [^177^Lu]Lu-DO3A.Glu.(FAPI)_2_ in PC3-mice at 4, 24, 48, 72 and 96 h p.i., with blocking at 4 h p.i. **(C)** Ex vivo autoradiography of PC3-tumors. Immunohistochemistry on a 7 μm frozen **(D)** outer and **(E)** inner PC3-tumor slice

PET/CT images of PC3-mice (Fig. 5A) demonstrate consistent tumor retention with low background uptake, aligning with the biodistribution data. SPECT/CT images (Fig. 5B) also match the biodistribution data, with slightly elevated background at 4 h p.i., particularly in bones and salivary glands. The images clearly demonstrate significant tumor uptake across all time points, with [^177^Lu]Lu-DO3A.Glu.(FAPI)_2_ showing faster clearance compared to [^177^Lu]Lu-DOTAGA.Glu.(FAPI)_2_, at later time points. The blocking studies confirmed the specific binding of all four radioligands.

### Ex vivo autoradiography

Autoradiography analysis of the tumor sections revealed a heterogeneous distribution of radioactivity within the PC3-tumors (Fig. 5C). Most of the radioactivity was mainly localized at the tumor periphery, while deeper regions exhibited a diminished signal.

### Immunohistochemistry

Immunohistochemistry confirmed both murine (stromal) and human (PC3 cell-derived) FAP expression in PC3-tumor (Fig. 5D-E). Stroma was heterogeneously distributed, with fewer stromal cells in the deeper tumor regions (Fig. 5E).

### Dosimetry in mice

The mean nTACs for [^177^Lu]Lu-DOTAGA.Glu.(FAPI)_2_ and [^177^Lu]Lu-DO3A.Glu.(FAPI)_2_ in heart content, liver, kidneys, salivary glands, bones, pancreas, lung, small intestine and tumor are presented in Fig. 4D. They demonstrate a progressive decline in activity over time due to the physical decay and biological clearance. Organs Like heart contents, lungs, and small intestine exhibit rapid initial washout. Tumor effective half-lives are 39.1 ± 1.1 h for [Lu]Lu-DOTAGA.Glu.(FAPI)_2_ and 51.1 ± 12.6 h for [^177^Lu]Lu-DO3A.Glu.(FAPI)_2_. [^177^Lu]Lu-DOTAGA.Glu.(FAPI)_2_ has longer effective half-lives in non-tumoral organs relative to [^177^Lu]Lu-DO3A.Glu.(FAPI)_2_; liver (198.8 ± 15.3 h vs. 70.4 ± 2.9 h), kidneys (50.1 ± 3.1 h vs. 22.3 ± 7.1 h) and salivary glands (52.1 ± 14.9 h vs. 11.8 ± 5.9 h), suggesting different pharmacokinetics and clearance. The liver’s effective half-life from the mono-exponential fit exceeded lutetium-177’s physical half-life (160 h), therefore it was Limited to 160 h.

TIACs/g (Table 2) show similar residual body activity (~ 11 h/g) for both, with liver and bones having the highest values. Liver TIAC/g for [^177^Lu]Lu-DOTAGA.Glu.(FAPI)_2_ is 2.7 times higher than for [^177^Lu]Lu-DO3A.Glu.(FAPI)_2_, indicating higher accumulation and retention.

**Table 2:** Murine and human tiacs/g for [^177^Lu]Lu-DOTAGA.Glu.(FAPI)_2_ and [^177^Lu]Lu-DO3A.Glu.(FAPI)_2_

	[^177^Lu]Lu-DOTAGA.Glu.(FAPI)_2_			[^177^Lu]Lu-DO3A.Glu.(FAPI)_2_		
	Mouse	Human		Mouse	Human	
		Male	Female		Male	Female
Heart contents	0.11 ± 0.005	0.09 ± 0.004	0.08 ± 0.004	0.12 ± 0.003	0.10 ± 0.002	0.09 ± 0.002
Kidneys	0.39 ± 0.02	0.13 ± 0.005	0.14 ± 0.006	0.20 ± 0.01	0.07 ± 0.002	0.07 ± 0.003
Liver	9.65 ± 0.82	3.30 ± 0.28	3.12 ± 0.26	3.47 ± 0.14	1.19 ± 0.05	1.12 ± 0.05
Pancreas	0.29 ± 0.01	0.04 ± 0.002	0.04 ± 0.002	0.20 ± 0.01	0.03 ± 0.001	0.03 ± 0.001
Salivary glands	0.36 ± 0.01	0.10 ± 0.003	0.10 ± 0.003	0.25 ± 0.02	0.07 ± 0.007	0.07 ± 0.007
Bones	6.65 ± 0.39	5.55 ± 0.33	4.90 ± 0.29	3.82 ± 0.12	3.18 ± 0.1	2.82 ± 0.09
Rest of the body	10.75 ± 0.68	15.06 ± 2	18.61 ± 2.6	10.77 ± 0.32	15.50 ± 0.7	16.80 ± 0.91
Tumor	1.19 ± 0.16	-	-	1.61 ± 0.2	-	-

### Extrapolated human dosimetry

Human TIACs/g are presented in Table 2 and S5-6. In adults, [^177^Lu]Lu-DOTAGA.Glu.(FAPI)_2_ showed highest TIACs/g in liver and bones. Murine-derived extrapolated TIACs predicted human dosing (Table 3), revealing generally higher absorbed doses (Gy/GBq) for [^177^Lu]Lu-DOTAGA.Glu.(FAPI)_2_ than [^177^Lu]Lu-DO3A.Glu.(FAPI)_2_, especially in liver (0.16–0.20 vs. 0.06–0.07), kidneys (0.04–0.05 vs. 0.02–0.03), and salivary glands (0.10–0.13 vs. 0.07–0.09). Osteogenic ce received the highest dose. The total body and effective doses were similar, though slightly higher for [lls^177^Lu]Lu-DOTAGA.Glu.(FAPI)_2_ (0.033–0.044 vs. 0.027–0.034). Given rising interest in ^161^Tb-labeled agents [36], dosimetry for [^161^Tb]Tb-DOTAGA.Glu.(FAPI)_2_ and [^161^Tb]Tb-DO3A.Glu.(FAPI)_2_ (Table 4) showed ~ 38% higher tissue doses vs. the ^177^Lu-labeled analogs. Detailed absorbed doses data are in Tables S7–14 (ICRP 89 male/female models).

**Table 3:** Target organ absorbed dose for [^177^Lu]Lu-DOTAGA.Glu.(FAPI)_2_ and [^177^Lu]Lu-DO3A.Glu.(FAPI)_2_

Average human absorbed dose extrapolations (mGy/Mbq)					
	[^177^Lu]Lu-DOTAGA.Glu.(FAPI)_2_		[^177^Lu]Lu-DO3A.Glu.(FAPI)_2_		
	M	F		M	F
Kidneys	4.08E-02 ± 0.002	4.97E-02 ± 0.002		2.09E-02 ± 0.001	2.54E-02 ± 0.001
Liver	1.64E-01 ± 0.01	2.00E-01 ± 0.02		6.00E-02 ± 0.002	7.30E-02 ± 0.003
Pancreas	3.11E-02 ± 0.002	3.90E-02 ± 0.002		2.12E-02 ± 0.001	2.62E-02 ± 0.001
Salivary glands	1.03E-01 ± 0.004	1.26E-01 ± 0.004		7.27E-02 ± 0.01	8.81E-02 ± 0.01
Osteogenic Cells	9.26E-02 ± 0.01	8.92E-02 ± 0.01		6.64E-02 ± 0.002	6.24E-02 ± 0.002
Red marrow	2.38E-02 ± 0.002	3.24E-02 ± 0.003		2.43E-02 ± 0.001	3.06E-02 ± 0.001
Spleen	2.87E-02 ± 0.01	3.53E-02 ± 0.01		5.27E-02 ± 0.002	6.42E-02 ± 0.003
Lungs	3.74E-02 ± 0.003	4.59E-02 ± 0.004		3.40E-02 ± 0.01	4.16E-02 ± 0.01
Small intestine	4.17E-02 ± 0.01	5.84E-02 ± 0.02		3.27E-02 ± 0.002	4.29E-02 ± 0.003
Total Body	3.25E-02 ± 0.003	4.42E-02 ± 0.01		2.71E-02 ± 0.001	3.44E-02 ± 0.002

**Table 4:** Target organ absorbed dose extrapolated for [^161^Tb]Tb-DOTAGA.Glu.(FAPI)_2_ and [^161^Tb]Tb-DO3A.Glu.(FAPI)_2_

Average human absorbed dose extrapolations (mGy/Mbq)						
	[^161^Tb]Tb-DOTAGA.Glu.(FAPI)_2_			[^161^Tb]Tb-DO3A.Glu.(FAPI)_2_		
	M	F	M		F	
Kidneys	5.60E-02 ± 0.002	6.83E-02 ± 0.003	2.84E-02 ± 0.001		3.48E-02 ± 0.001	
Liver	2.29E-01 ± 0.02	2.78E-01 ± 0.02	8.31E-02 ± 0.003		1.01E-01 ± 0.004	
Pancreas	4.25E-02 ± 0.002	5.34E-02 ± 0.003	2.88E-02 ± 0.001		3.59E-02 ± 0.001	
Salivary glands	1.43E-01 ± 0.01	1.73E-01 ± 0.01	1.00E-01 ± 0.01		1.22E-01 ± 0.01	
Osteogenic Cells	1.24E-01 ± 0.01	1.23E-01 ± 0.01	8.97E-02 ± 0.003		8.70E-02 ± 0.003	
Red marrow	3.37E-02 ± 0.003	4.59E-02 ± 0.01	3.45E-02 ± 0.001		4.37E-02 ± 0.002	
Spleen	3.92E-02 ± 0.01	4.83E-02 ± 0.01	7.27E-02 ± 0.003		8.87E-02 ± 0.004	
Lungs	5.19E-02 ± 0.004	6.37E-02 ± 0.01	4.73E-02 ± 0.01		5.78E-02 ± 0.01	
Small intestine	5.69E-02 ± 0.02	4.17E-02 ± 0.01	2.85E-02 ± 0.001		3.72E-02 ± 0.002	
Total Body	4.51E-02 ± 0.01	6.01E-02 ± 0.01	3.70E-02 ± 0.002		4.70E-02 ± 0.002	

## Discussion

FAPI-based radiopharmaceuticals show promise as theranostics, however, they require improvements to enhance their therapeutic efficacy [6, 7, 9, 19, 26, 37–39] since they clear rapidly from tumors, limiting their effectiveness. Our prior preclinical [12, 16] and clinical [6, 7, 21, 22, 26] data emphasized the need to optimize [^177^Lu]Lu-DOTAGA.(SA.FAPI)_2_ pharmacokinetics. In response, the second generation of FAPI homodimers, DOTAGA.Glu.(FAPI)_2_ and DO3A.Glu.(FAPI)_2_, was prepared [15].

Saturation binding [12] and enzymatic assays (Table S15) [3, 15, 16] demonstrated that the SA-to-Glu substitution enhanced the Glu-homodimers affinity. While direct comparison is not possible, affinity trends are consistent. ^68/nat^Ga-DOTAGA.(SA.FAPI)_2_ and ^177/nat^Lu-DOTAGA.(SA.FAPI)_2_ showed K_d_ of 1.15 ± 0.26 nM and 1.35 ± 0.69 nM, respectively [12]. However, ^68/nat^Ga-DOTAGA.Glu.(FAPI)_2_, ^68/nat^Ga-DO3A.Glu.(FAPI)_2_, ^177/nat^Lu-DOTAGA.Glu.(FAPI)_2_, and ^177/nat^Lu-DO3A.Glu.(FAPI)_2_, exhibited improved affinities (K_d_:0.7–0.9 nM). Similarly, the enzymatic assays showed IC_50_ of 0.9 nM (DOTAGA.(SA.FAPI)_2_) and 1.5 nM (^nat^Lu-DOTAGA.(SA.FAPI)_2_), while their Glu-containing versions exhibited IC_50_ of ~ 0.3 nM. The SA-to-Glu substitution likely enhances radioligand-receptor interaction by introducing carboxyl groups, improving the conformational stability, FAP active site binding, and receptor accessibility.

Lipophilicity and plasma protein binding are key parameters in predicting the in vivo performance of radioligands, as they strongly influence systemic clearance, target tissue penetration and retention, as well as non-specific background signal. These properties are commonly assessed early in radioligand development to estimate in vivo suitability, but must be considered in balance with other molecular features, such as metabolic stability, polarity, and receptor affinity, to achieve optimal imaging or therapeutic performance. LogD and protein binding data (Table S16) also highlight that structural changes affect their hydrophilic character. [^68^Ga]Ga-DOTAGA.(SA.FAPI)_2_ and [^177^Lu]Lu-DOTAGA.(SA.FAPI)_2_ had moderate lipophilicity (LogD: −1.8 ± 0.02 and − 1.7 ± 0.03, respectively) [12, 16]. Functionalization with the DO3A chelator of the Glu-containing FAPIs maintained the lipophilicity: −2.2 ± 0.04 for [^68^Ga]Ga-DO3A.Glu.(FAPI)_2_ and − 1.7 ± 0.01 for [^177^Lu]Lu-DO3A.Glu.(FAPI)_2_. Functionalization with DOTAGA in DOTAGA.Glu.(FAPI)_2_ lowered LogD to −2.9 ± 0.1 (gallium-68) and − 3.0 ± 0.1 (lutetium-177), reflecting increased polarity from the added polar groups. Protein binding decreased accordingly: the SA-containing radioligands [12] bound to plasma proteins at 18 ± 1.1% (gallium-68) and 25.3 ± 0.8% (lutetium-177), while the Glu-containing analogs showed reduced binding of 9.4 ± 2.2% and 9.2 ± 0.03%, respectively.

In animal studies, [^177^Lu]Lu-DOTAGA.(SA.FAPI)_2_ was predominantly eliminated via the hepatobiliary route [12], whereas [^177^Lu]Lu-DOTAGA.Glu.(FAPI)_2_ showed increased renal clearance. A clinical case also supported these findings [15]. After two treatment cycles with [^177^Lu]Lu-DOTAGA.(SA.FAPI)_2_, and a third using [^177^Lu]Lu-DOTAGA.Glu.(FAPI)_2_, imaging showed that [^177^Lu]Lu-DOTAGA.(SA.FAPI)_2_, despite effective lesion uptake, exhibited high Liver uptake at 24 h and colon uptake at 48 h. [^177^Lu]Lu-DOTAGA.Glu.(FAPI)_2_ had similar tumor uptake but reduced liver and colon accumulation. These findings highlight how tuning lipophilicity and protein binding through rational structural modifications can modulate clearance pathways and reduce off-target accumulation, improving imaging contrast and therapeutic specificity.

Our study on circulating FAP highlighted the importance of precise dosing for optimal in vivo performance [35]. Due to higher lipophilicity of [^177^Lu]Lu-DO3A.Glu.(FAPI)_2_, we reduced the previous optimum injected mass from 600 to 450 pmol, while keeping [^177^Lu]Lu-DOTAGA.Glu.(FAPI)_2_ at 600 pmol. Both exhibited similar liver (~ 1.7%I.A./g) and comparable tumor uptake at 4 h p.i. However, [^177^Lu]Lu-DOTAGA.Glu.(FAPI)_2_ showed superior tumor residency, with slightly higher uptake at all time points, > 2%IA/g even at 96 h, higher tumor-to-blood ratios, and longer tumor half-life (25.5 h) (Fig. S7, Table S4). Compared to [^177^Lu]Lu-DOTAGA.(SA.FAPi)_2_ (~ 8%I.A./g), [^177^Lu]Lu-DOTAGA.Glu.(FAPI)_2_ and [^177^Lu]Lu-DO3A.Glu.(FAPI)_2_ exhibited significantly higher tumor uptake (~ 15%I.A./g) at 1 h p.i. Despite differences in initial uptake, all three had similar clearance: ~50% washed-out by 24 h. The first generation, [^177^Lu]Lu-DOTAGA.(SA.FAPi)_2_, due to its lipophilicity, showed high non-specific uptake (liver, spleen, kidneys, pancreas, bones) that persisted up to 96 h [12]. These drawbacks were addressed in the improved pharmacokinetics of [^177^Lu]Lu-DOTAGA.Glu.(FAPI)_2_ and [^177^Lu]Lu-DO3A.Glu.(FAPI)_2_. Additionaly, all dimers outperformed the fast-clearing monomer [^177^Lu]Lu-DOTA.SA.FAPI, which was nearly undetectable by 4 h p.i [12].

To evalaute tumor uptake in the low FAP-expressing PC3-model, we investigated murine fibroblasts recruitment, which creates heterogeneous FAP distribution. DOTAGA.Glu.(FAPI)_2_ and DO3A.Glu.(FAPI)_2_ showed uptake of ~ 20% (gallium-68) and ~ 16% (lutetium-177), indicating strong binding, likely from the FAP-positive stroma. This supports prior studies that murine FAP (89% identical to human FAP) contributes similarly to tumor progression [40]. Our immunohistochemistry data confirmed dual FAP expression, validating dual targeting with FAPI-radioligands in preclinical models. Autoradiography showed heterogeneous FAP expression, with higher levels at tumor margins and lower in the core.

Direct comparison with other preclinical studies is challenging due to variations in tumor models, study designs, and experimental conditions. Differences in tumor biology, stromal content, and FAP expression can affect radioligand behavior, complicating efficacy interpretation and potentially obscuring structure-activity relationships. Standardizing preclinical protocols and models would enhance comparability and support the translational development of FAPI radioligands.

Preclinical dosimetry studies are vital for assessing radiation dose distribution, predicting efficacy and toxicity, and optimizing dosing of new radiopharmaceuticals. Extrapolating to humans is a complex process due to interspecies differences in metabolism, biodistribution, and clearance [31]. In preclinical and early clinical studies [41], [^177^Lu]Lu-DOTAGA.Glu.(FAPI)_2_, showed rapid tumor and target organs uptake within one hour. Its biological half-life was 25.5 h in PC3-tumors, while in human studies it was 22.1 ± 6.7 h in soft tissue metastases, and 19.5 ± 7.3 h in bone metastases. Human dosimetry identified the colon as the critical organ receiving the highest absorbed radiation dose, with mean values of 0.31 ± 0.12 Gy/GBq for the left colon and 0.22 ± 0.09 Gy/GBq for the right colon [41]. A direct comparison with preclinical data was not possible for the colon, as colon was not included in the murine biodistribution studies. Previous human dosimetry data for the first generation FAPI-dimeric radioligand [^177^Lu]Lu-DOTAGA.(SA.FAPi)_2_ (32) also highlighted the colon as the dose-limiting organ, though with substantially higher absorbed doses (right colon: 1.16 ± 0.86 Gy/GBq; left colon: 2.87 ± 1.74 Gy/GBq) than those observed for [^177^Lu]Lu-DOTAGA.Glu.(FAPI)_2_. The clinical dosimetry data on [^177^Lu]Lu-DOTAGA.Glu.(FAPI)_2_ [41], showed, that he organs typically classified as organs at risk receive radiation doses well below their critical thresholds. Nevertheless, the relatively short retention times at the tumor sites, particularly when compared to PSMA [42] and SSTR [43] radioligands, restrict the maximum deliverable tumor doses.

Due to their similar physical half-lives (lutetium-177: 6.65 days; terbium-161: 6.96 days), comparable β^−^ emissions, and nearly identical coordination chemistry, it is scientifically justified to extrapolate terbium-161 biodistribution and macroscopic dosimetry from ^177^Lu-labeled radioligands, assuming identical biological behavior. While terbium-161 emits additional low-energy Auger and conversion electrons that enhance therapeutic efficacy at the cellular level, these do not influence organ-level distribution. Thus, lutetium-177 biodistribution data serve as a valid surrogate for terbium-161in organ-level dosimetry, with microscale dose contributions considered separately. The ^161^Tb-labeled radioligands deliver ~ 38% higher tissue doses than ^177^Lu-labeled ones due to higher β⁻ energy and Auger electron emission, potentially enhancing efficacy. Dosimetric analysis (excluding the colon) identifies red bone marrow as the dose-limiting organ for both, with marrow toxicity (~ 2 Gy threshold) reached at ~ 65 GBq (lutetium-177) and ~ 45 GBq (terbium-161). At these doses, liver absorption is ~ 10 Gy, with a tumor-to-liver TIAC/g ratio of ~ 2.3, predicting ~ 25 Gy to tumors. Faster tumor clearance may reduce efficacy, suggesting best use in highly radiosensitive cancers. Despite promising results from a single tumor model, broader preclinical validation across multiple tumor models and human trials are essential.

In conclusion, the development of the second-generation Glu-containing FAPI dimers represents a substantial advancement in FAPI-targeted radiopharmaceuticals, showing better pharmacokinetics, higher tumor uptake and retention, lower off-target accumulation, and reduced nonspecific binding. [^177^Lu]Lu-DOTAGA.Glu.(FAPI)_2_ stands out with superior biodistribution and dosimetry over the first-generation versions. These results support further clinical evaluation across diverse FAP-expressing cancers.

Significant differences at:

−48 h (tumor *p* < 0.0001, liver *p* = 0.0046, salivary glands *p* = 0.0002),
−72 h (tumor *p* < 0.0001, liver *p* = 0.0002, kidney *p* = 0.03, bone *p* = 0.002, salivary glands *p* = 0.025),
−96 h (tumor *p* < 0.0001, liver *p* < 0.0001, kidney *p* = 0.0003, bone *p* < 0.0001, salivary glands *p* < 0.0001).

## Supplementary Information

Below is the link to the electronic supplementary material.

Supplementary Material 1 (DOCX 535 KB)
