# Supplementary material for: Preclinical evaluation of DOTAGA.Glu.(FAPI)2 and DO3A.Glu.(FAPI)2 as theranostics with human dosimetry extrapolation to lutetium-177 and terbium-161 analogs
Source: Eur J Nucl Med Mol Imaging. 2025 Oct 17;53(3):1887–901. doi: 10.1007/s00259-025-07565-9 (PMC12860872; doi:10.1007/s00259-025-07565-9)
Supplement: Supplementary file 1 — Supplementary Material 1 (DOCX 535 KB) [file 259_2025_7565_MOESM1_ESM.docx]

**Supplemental Data**

**Preclinical evaluation of DOTAGA.Glu.(FAPI)_2_ and DO3A.Glu.(FAPI)_2_ as theranostics with human dosimetry extrapolation to lutetium-177 and terbium-161 analogs**

Adrianna Bilinska^1,2^, Naveen Kumar^1^, Silvano Gnesin^3^, Tilman Läppchen^1^, Elena Menéndez^1^, Marcel Martin^4^, Frank Rösch^4^, Axel Rominger^1^, Eleni Gourni^1^

^1^Department of Nuclear Medicine, Inselspital, Bern University Hospital, Switzerland,

^2^Graduate School of Cellular and Biomedical Sciences, University of Bern, Bern, Switzerland

^3^Institute of Radiation Physics, Lausanne University Hospital and University of Lausanne, Lausanne, Switzerland

^4^Department of Chemistry–TRIGA site, Johannes Gutenberg-University Mainz, Germany

**MATERIAL AND METHODS**

**General**

All reagents were of the best grade available and were purchased from common suppliers. All culture reagents were from Gibco BRL, Life Technologies (Grand Island, NY). The human prostate adenocarcinoma cell line PC3 (CLS-300312, Lot number 816SF) and cancer associated prostate fibroblast cell line (hTERT PF179T, ATCC CRL-3290, Lot Number 0303) were obtained from Cell Lines Service GmbH (CLS, Eppelheim, Germany) and the American Type Culture Collection (ATCC, Manassas, Virginia), respectively. The human serum was commercially available by Sigma (H4522). [^177^Lu]LuCl3 in 0.04 M HCl was obtained from DSD-Pharma GmbH (Purkersdorf near Vienna, Austria). The GalliaPharm® Ge-68/Ga-68 generator was available from Eckert & Ziegler (Berlin, Germany). The quality control of the precursor and the radiotracers was performed by an analytical Reverse-Phase High Performance Liquid Chromatography (RP-HPLC) on an analytical Nucleosil 120-5 column C18 (250 x 4.5 mm) applying a linear gradient of 15-90% solvent B in 30 min at a flow rate of 1 mL/min. (solvent A, 0.1% trifluoroacetic acid (TFA)/water (H2O); solvent B, 0.1%TFA/acetonitrile (ACN). Ultraviolet detection was performed using an Agilent detector at 214 nm. For radioactivity measurement, a Na(TI) well-type scintillation Gina star was used. The radiotracer solutions for the experiments were prepared by dilution with 0.9% NaCl (Bichsel AG, Interlaken, Switzerland).

Quantitative γ-counting was performed with a COBRA II γ-system well counter from Packard Instrument (USA). For μPET/CT studies, a dedicated micro-PET/SPECT/CT scanner (Albira Si; Bruker Biospin, Ettlingen, Germany) was used.

Mice were purchased from Janvier Labs (Rue du Genest, 53940 Le Genest-Saint-Isle, France), pentobarbital natrium (150mg/kg) from Streuli Pharma SA (Uznach, Switzerland).

PC3 tumors xenografts aimed for immunohistochemistry and autoradiography were snap frozen in liquid nitrogen and then cut in Leica cryostat to obtain 7 um sections.

**Radiochemistry / Quality control of the radiotracers**

**Radiolabeling with gallium-68**

[^68^Ga]Ga-DOTAGA.Glu.(FAPI)_2_ and [^68^Ga]Ga-DO3A.Glu.(FAPI)_2_ were prepared within 5 min, using the Modular-Lab PharmTracer module by Eckert&Ziegler (Berlin, Germany). The radiolabeling performance of all precursors was assessed at pH 4.6 with 1 M HEPES buffer at 90 °C for 5 min using, in each case, the minimum conjugate amount. Briefly, the Ge-68/Ga-68 generator was eluted with 5 mL 0.1 N HCl and the eluate (~300 MBq) was loaded onto a cation exchange column (Strata-XC, Phenomenex). Gallium-68 was eluted with 700 μL of a mixture of 5.5 M NaCl / 0.1 M HCl (prepared by mixing 0.53 mL of ultrapure 30% aq. HCl and 49.5 mL of 5.5 M NaCl) directly into a vial containing 2 mL 1 M HEPES (pH 5.3), 200 μL of EtOH, and 20 μg (approximately 20 nmol) of the conjugate, followed by SepPak C-18 purification to remove uncomplexed gallium-68. The purified [^68^Ga]Ga-DOTAGA.Glu.(FAPI)_2_ and [^68^Ga]Ga-DO3A.Glu.(FAPI)_2_ were eluted in 1.4 mL of a solution of 6.2% EtOH in PBS.

The quality control of [^68^Ga]Ga-DOTAGA.Glu.(FAPI)_2_ and [^68^Ga]Ga-DO3A.Glu.(FAPI)_2_ was performed by radio-HPLC as described in the reagents and instrumentation. The presence of free gallium-68 and ^68^Ga-labelled colloid in the [^68^Ga]Ga-DOTAGA.Glu.(FAPI)_2_ and [^68^Ga]Ga-DO3A.Glu.(FAPI)_2_ preparations was quantified by radio thin layer chromatography (Radio-TLC) using ITLC-SG-plates (Glass microfiber chromatography paper impregnated with silica gel) and two different mobile phase eluents: a) 0.1 M Na-citrate; b) MeOH / 1 M ammonium acetate (1/1, v/v).

**Radiolabeling with lutetium-177**

[^177^Lu]Lu-DOTAGA.Glu.( FAPI)_2_ and [^177^Lu]Lu-DO3A.Glu.( FAPI)_2_ were prepared by dissolving 6.5 μg (approximately 5 nmol) of DOTAGA.Glu.(FAPI)_2_ and DO3A.Glu.( FAPI)_2_ respectively in 200-250 μL HEPES buffer (1 M, pH 5.3), followed by incubation with [^177^Lu]Lu^3+^ (30 - 200 MBq) at 90 °C for 30 min. Both products were used without any further purification step.

The quality control of [^177^Lu]Lu-DOTAGA.Glu.(FAPI)_2_ and [^177^Lu]Lu-DO3A.Glu.(FAPI)_2_ was performed by radio-HPLC and radio thin layer chromatography (radio-TLC) as described in the reagents and instrumentation.

After the labelling with gallium-68 or lutetium-177 and the quality control of the generated radiotracers, one equivalent of either ^nat^Ga(NO_3_)_3_ or ^nat^LuCl_3_ x 6H_2_O were added to the relevant radiolabelling solutions. The final solutions were incubated at 90 °C for 30 min to obtain structurally characterized homogeneous ligands which were used for the saturation binding studies. The homogeneity was determined by HPLC, showing one peak.

**Lipophilicity and protein binding studies**

The lipophilicity (LogD_octanol/PBS,_ pH 7.4) of [^68^Ga]Ga-DOTAGA.Glu.(FAPI)_2,_ [^68^Ga]Ga-DO3A.Glu.(FAPI)_2,_ [^177^Lu]Lu-DOTAGA.Glu.(FAPI)_2_ and [^177^Lu]Lu-DO3A.Glu.(FAPI)_2_ was estimated by the “shake-flask” method: The radiolabelled conjugates (20 pmol; 0.3 MBq) were added to a 1:1 mixture of 1-octanol (500 μL) and PBS (500 μL, pH 7.4). The mixture was intermittently vortexed for 1 hour to reach the equilibrium and then centrifuged (3000 rpm) for 10 minutes. From each phase, an aliquot (100 μL) was pipetted out and measured in a gamma-counter. Each measurement was repeated five times. Care was taken to avoid cross-contamination between the phases. The partition coefficient was calculated as the average log ratio of the radioactivity in the organic fraction and the PBS fraction.

[^68^Ga]Ga-DOTAGA.Glu.(FAPI)_2_, [^68^Ga]Ga-DO3A.Glu.(FAPI)_2_ (~120 pmol; ~2 MBq) and [^177^Lu]Lu-DOTAGA.Glu.(FAPI)_2_ and [^177^Lu]Lu-DO3A.Glu.(FAPI)_2_ (~70 pmol; 0.14 MBq) were incubated with commercially available human serum (0.5 mL) at 37 °C for 30 minutes. When the incubating period was completed, proteins were precipitated with a solution of 1 mL MeOH/ACN (1:1). Centrifugation (10 minutes, 3000 rpm) was performed for the separation of proteins. After careful separation of the two phases, the respective activities were measured in a gamma-counter, followed by determination of the percentage of the radiotracer which binds to the serum proteins.

**Cell lines**

PC3 cell lines were cultured in Dulbecco's Modified Eagle Medium (DMEM) with low glucose (1g/L) and F-12 Nutrient Mix with GlutaMAX™-I in a 1:1 mixture ratio. The CAFs were cultured using the Eagle's Minimal Essential Medium (EMEM) supplemented with sodium bicarbonate 1500 mg/L and puromycin 1 μg/mL. The cultivation media were supplemented with 5% fetal bovine serum for PC3 cells and 10% for CAF cells, along with penicillin (100 U/mL) and streptomycin (100 µg/mL). Both cell lines were cultured at 37°C and 5% CO_2_.

All the cells lines were purchased from commercial suppliers and they are accompanied by certificates of analysis that verify their compliance with pathogen-free standards. Furthermore, each cell line undergoes internal testing within the laboratory on an annual basis to ascertain their freedom from mycoplasma contamination.

**Saturation binding studies**

For receptor saturation analysis, the CAF cells overexpressing FAP were seeded at a density of 0.8-1 million cells per well in 6-well plates, pre-coated with BME (Cultrex Reduced Growth Factor Basement Membrane Extract, RGF BME, R&D System, Minneapolis, USA) to enhance cell attachment. The cells were incubated overnight with medium (EMEM supplemented and containing 10% FBS, 100 U/mL penicillin and 100 μg/mL streptomycin). The next day, the medium was removed and the cells were incubated for 30 minutes at 37 °C, with 0.8 mL of fresh medium. Afterwards, the plates were placed on ice for 30 minutes followed by incubation with increasing concentrations of ^68/nat^Ga- and ^177/nat^Lu-labelled DOTAGA.Glu.(FAPI)_2_ and DO3A.Glu.( FAPI)_2_ (0.1-10 nM final concentration in the wells). After the addition of the radioligands, the cells were incubated for 120 minutes at 4 °C. Non-specific binding was determined in the presence of UAMC1110 at a final concentration of 1 μM. After the completion of the incubation, the cells were washed twice with ice-cold PBS, followed by solubilization with 1 M NaOH. The cell-associated radioactivity was measured using a gamma-counter. Specific binding was plotted against the total molar concentration of the added radiotracer. The K_d_ values and the concentration of the radioligand required to saturate the receptors (B_max_) were determined by nonlinear regression using GraphPad (Prism 8 Graph Pad Software, San Diego, CA). For all the cell studies the values are normalized for 1x10^6^ cells per well and all data are from two independent experiments with triplicates in each experiment.

**Internalization studies**

For internalization experiments, CAF cells were seeded into 6-well plates pre-coated with BME and treated as described at the saturation binding studies. On the day of the experiment, approximately 2.5 pmol (100 µL) of the radiotracer was added to the medium (total volume 1.5 mL) and the cells were incubated (in triplicates) for 15, 30, 60, 90, 120, 180 and 240 minutes at 37 °C, 5% CO_2_ for the ^68^Ga-labeled radioligands and for 0.5, 1, 2, 4 and 6 hours at 37 °C, 5% CO_2_ for the ^177^Lu-labeled radioligands. To determine nonspecific membrane binding and internalization, excess of UAMC1110 (final concentration 1 μΜ) was added to selected wells. At each time point, the internalization was stopped by putting the plates on ice, removing the medium and washing the cells twice with ice-cold PBS. To remove the membrane-bound radioligand, an acid wash was carried out twice with a 0.1 M glycine buffer pH 2.8 for 5 minutes on ice. Finally, cells were solubilized with 1 M NaOH. The radioactivity of the culture medium, the membrane-bound, and the internalized fractions were measured in a gamma-counter.

**Externalization studies**

The externalization rate of [^177^Lu]Lu-DOTAGA.Glu.(FAPI)_2_ and [^177^Lu]Lu-DO3A.Glu.(FAPI)_2_ was studied after CAFs were incubated for 2 hours with 2.5 pmol/well of each ^177^Lu-lebeled radioligand. After the completion of the 2 hours of incubation, the medium was removed and CAF cells were washed with 1 mL of cold PBS (x2). The cells were then exposed to an acid wash with glycine buffer, as described above, to dissociate membrane-bound radioligand, and the fractions were collected. Fresh medium (1 mL/well) was added to the cells and were incubated at 37 °C. At different time points (10, 20, 30, 60, 120, 240 and 1440 minutes) the external medium was removed (followed by two washing steps with cold PBS). Finally, cells were solubilized with 1 M NaOH. The radioactivity of the culture medium, the membrane-bound, the internalized and the externalized fractions were measured in a gamma-counter. The externalized fraction of radioactivity was expressed as the percentage of the total internalized amount.

**Animal models**

For the imaging and biodistribution experiments, mice were randomly designated to groups based on their tumor sizes to ensure that each group had a similar size distribution.

**Metabolic stability**

Approximately 1 mL of blood from PC3-mice was collected to heparinized tubes at 30 minutes after intravenous injection of 600 pmol of [^177^Lu]Lu-DOTAGA.Glu.(FAPI)_2_ and 450 pmol of [^177^Lu]Lu-DO3A.Glu.(FAPI)_2_ (in a total injected volume of 100 µL of 0.9% NaCl) (n=3). Following blood collection, plasma was separated by centrifugation at maximum speed for 10 minutes at 4 ºC. The plasma was then treated with a 1:1 solution of methanol and acetonitrile to precipitate the proteins. After centrifugation at maximum speed for 10 minutes at 4 ºC, 60 µL of the supernatant were injected into RP-HPLC.

**Small-animal PET/SPECT/CT imaging**

PET/CT scans were acquired using a dedicated micro-PET/SPECT/CT scanner (Albira Si; Bruker Biospin, Ettlingen, Germany).PET images were corrected for gallium-68 decay and reconstructed with 1 iteration of Ordered Subset Expectation Maximization (OSEM) algorithm using a voxel size of 0.75 mm. Partial Volume correction along with a Point Spread Function iterative deconvolution were applied. The images were normalized and generated using PMOD software. The color scale of the PET images was set from 0 to 12 % I.A./mL allowing for qualitative comparison among the images. The CT was carried out using step and shoot mode and employed 45 kVp and 400 μA as settings. The CT data were reconstructed using Filtered Back Projection (FBP) algorithm and a voxel size of 0.125 mm.  The color scale of the CT is ranging from 150 to 700 Hounsfield Units (HUs).

SPECT images were obtained after 4, 24, 48, 72 and 96 hours upon injection of 600 pmol (~13 MBq/100 μL) of [^177^Lu]Lu-DOTAGA.Glu.(FAPI)_2_ and 450 pmol (~11 MBq/100 μL) of [^177^Lu]Lu-DO3A.Glu.(FAPI)_2_ in PC3-mice.

Blocking studies were conducted in the same way as the biodistribution studies. For the blocking studies, the PET/CT images were acquired at 1 hour p.i., while the SPECT/CT images were obtained at 4 hours p.i..

SPECT images were acquired using a 208 keV ± 20 % energy window and a Cerrobase (Bi-Pb alloy) 30 mm thick collimator, with a pinhole 22 mm thick tungsten lens, 60 projections and 180 – 1440 s per projection. SPECT data were reconstructed using the Ordered Subset Expectation Maximization (OSEM) algorithm with 2 iterations of 5 subsets and a voxel size of 0.5 mm. The reconstructed data were corrected for lutetium-177 decay, normalized and filtered, using a Gaussian 3D algorithm with a 1.5 mm isotropic kernel, and generated using PMOD software. The color scale of the SPECT images was set from 0 to 16 % I.A./mL, allowing for qualitative comparison among the images. The CT was carried out using step-and-shoot mode, employing 45 kVp and 400 μA as settings. CT data were reconstructed using FBP algorithm and a voxel size of 0.125 mm. The color scale of the CT is ranging from -100 to 700 HUs.

**Immunohistochemistry**

Immunohistochemical staining was conducted using frozen tumor sections (7 µm) from the same tumors used for autoradiography, with consecutive sections taken directly from the autoradiography samples.. The tumor sections were washed three times in phosphate-buffered saline and then fixed with 4% paraformaldehyde for 10 minutes. Following an additional washing step, the sections were permeabilized for 10 minutes, and a blocking solution containing 2% horse serum was applied. The tumor sections were incubated overnight with 15 µg/mL of Mouse Fibroblast Activation Protein Antibody (R&D Systems, MAB9727-SP). The next day, they were washed once more and incubated overnight this time with 10 µg/mL of Human Fibroblast Activation Protein Antibody and 2.5 µg/mL of αSMA antibody (R&D Systems, AF3715). On the following day, the tumor sections underwent a washing step before the incubation with the secondary antibodies: Donkey anti-Rat IgG (H+L) Highly Cross-Adsorbed Secondary Antibody, Alexa Fluor™ 647 (ThermoFisher Scientific, A78947), Donkey anti-Sheep IgG (H+L) Cross-Adsorbed Secondary Antibody, Alexa Fluor™ 594 (ThermoFisher Scientific, A-11016) and Goat anti-Mouse IgG (H+L) Cross-Adsorbed Secondary Antibody, Alexa Fluor™ 488 (ThermoFisher Scientific, A-11001). All secondary antibodies were used at a 1:500 dilution and incubated for 1 hour at room temperature. After a final wash, DAPI Vectashield Antifade Mounting Media (Vector Laboratories, Switzerland) was applied to the tumor sections.

The stained sections were analyzed using a Zeiss LSM980 Confocal Microscop and Zen 3.4 Software.

**Dosimetry**

Biodistribution kinetics for [177Lu]Lu-DOTAGA.Glu.(FAPI)2 and [177Lu]Lu-DO3A.Glu.(FAPI)2 were assessed by ex vivo quantification of the accumulated radioactivity in source organs using a calibrated gamma counter at 4, 24, 48, 72, and 96 hours p.i. (n=3). The percentage of injected activity per gram of tissue (%I.A./g) was calculated, as described above, for a comprehensive panel of tissues, including liver, lungs, stomach, spleen, pancreas, kidneys, intestines, bone, muscle, salivary glands, heart wall, blood, and tumor. To estimate the absolute activity in each source organ, the individual %I.A./g values was multiplied by the corresponding organ mass derived from the standard 25 g MOBY mouse phantom, which provides high-resolution anatomical reference data for the murine models (28). This approach resulted in the normalized source organ activities (nA) for each time point and animal. The actual activity measurements were referred to the gamma counter measurement time (i.e. no physical decay correction to the administration time was applied). For each organ and time point, the mean nA was subsequently calculated across the three animals.

As outlined by Cicone et al., (1) the nA values for source organs were corrected to account for the fraction of administered activity sequestered by the tumor by redistributing the tumor nA (nA_tumor_) to organs according to the formula:

$${nA}_{organ,corrected}= {nA}_{organ}+\left( {nA}_{tumor}\times\frac{{nA}_{organ}}{{nA}_{WB}-{nA}_{tumor}} \right)$$

Where nA_WB_ is the nA measured in the whole body. This adjustment was implemented to facilitate the extrapolation of absorbed dose data from mice to humans, considering that the tumor "sink effect" - prominent in murine models - is generally negligible in human subjects. In radiopharmaceutical dosimetry using tumor-bearing mice, the “tumor sink” effect refers to the phenomenon where large or highly avid tumors absorb a significant portion of the injected radiotracer, reducing its availability to normal organs. This can lead to artificially low uptake measurements in healthy tissues, which may not reflect what would occur in humans with smaller tumors or lower tumor burden. To correct for this, dosimetry calculations often include a "tumor sink correction" that adjusts normal organ uptake values to account for the radiotracer sequestered by the tumor, ensuring more accurate and clinically relevant dose estimates.

According to the MIRD formalism, for each source organ, time-integrated activity coefficients (TIACs) were derived by integrating the normalized time-activity curves (nTACs), which were fitted using the bi-exponential functions. The integration of the fitted nTACs was extended to infinity for organs in which the effective half-life of the slower bi-exponential component (tslow) was shorter than the physical half-life of lutetium-177 (tslow < tphys). In contrast, for organs exhibiting tslow > tphys a mono-exponential fit, considering the Lu-177 physical decay constant, was applied beyond the last measured time point. Additionally, the nTACs were analyzed to characterize and compare the temporal distribution profiles of the radioligands across the evaluated source organs, with particular attention to the effective and the biological half-lives.

TIACs were calculated by fitting the nTACs with the bi-exponential functions and performing time integration in MATLAB (R2021a, The MathWorks, Inc., Natick, MA, USA). Additionally, mass-normalized TIACs (TIAC/g) were derived using organ masses from the standard 25 g MOBY mouse phantom. These values were employed to evaluate the potential absorbed dose deliverable to the tumor relative to that in dose-limiting organs - such as the liver and red marrow - in the context of extrapolating murine dosimetry data to humans. TIAC for the analogue ^161^Tb radiolabeled compounds were obtained fitting the ^161^Tb nTACs, where:

$${nA}_{Tb-161}\left( t \right)={nA}_{Lu-177}\left( t \right)\times\left( e^{(ln \left( 2 \right)/\tau_{Lu-177})\times t} \right)\times\left( e^{(-ln \left( 2 \right)/\tau_{Tb-161})\times t} \right)$$

With $\tau_{Lu-177}$ = 6.65 h and $\tau_{Tb-161}$= 6.96 h being the physical half-lives of ^177^Lu and ^161^Tb respectively. In reason of the similar physical half-life of the two radiolabeled compounds the organ TIAC for the case of ^161^Tb never exceeded the corresponding organ TIAC for the ^177^Lu more than 1.5% (data not shown), therefore in this work we assumed organ TIAC_Tb-161_= organ TIAC_Lu-177_.

**Extrapolation of murine TIACs to human equivalents**

The TIACs, for [177Lu]Lu-DOTAGA.Glu.(FAPI)2 and [177Lu]Lu-DO3A.Glu.(FAPI)2, were determined by time integration of the nTACs. To account for the inter-animal variability, the TIACs for each source organ were calculated using both the mean nTACs and the nTACs obtainend from fitting the mean ±1 standard deviation (SD) of the normalized time-activity data. This approach provided the upper and the lower bounds for the estimated TIACs.

The mice-to-human TIAC extrapolation was obtained considering the different (species-specific) relative source organ masses, m(organ), as compared to the total body weight (WB), for the mice (m) and human (h) models respectively.


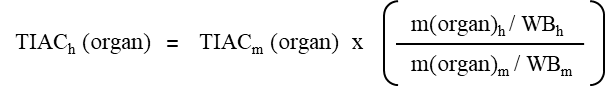


The human organ masses, m(organ)h, were obtained from the adult male and female reference models phantoms defined in ICRP-89, while the murine organ masses, m(organ)m, were derived from the standard MOBY mouse model. The extrapolated human TIACs values, TIACh, for each source organ were input into the kinetic module of the OLINDA/EXM software (version 2.1, HERMES Medical Solutions AB, Stockholm, Sweden) to compute the absorbed doses to target organs and the effective dose for adult male, female, and gender-averaged human models.

Additionally, human dosimetry for the same compounds was estimated assuming radiolabeling with terbium-161 instead of lutetium-177. Given the comparable physical half-lives of the two radionuclides (6.95 days for terbium-161 and 6.7 days for lutetium-177) and assuming identical biological distribution, we approximated TIAC_h,Tb-166_ = TIAC_h,Lu-177_. For this purpose, the decay data for terbium-161 were substituted for those of lutetium-177 within the OLINDA dosimetry calculations, while all other parameters remained unchanged.

**Statistical analysis**

A two-way ANOVA was used to assess differences in biodistribution between each pair of the [⁶⁸Ga]- and [¹⁷⁷Lu]-labelled radiopharmaceuticals across organs and timepoints. Analyses were performed using GraphPad Prism 8, with statistical significance set at P < 0.05 (95% confidence level). Multiple comparisons were corrected using the Sidak method, as recommended by the software. Due to the limited sample size (n = 3 per group), formal testing of data distribution was not feasible.

**REFERENCES**

1. Cicone, F., Denoël, T., Gnesin, S. *et al.* Preclinical Evaluation and Dosimetry of [^111^In]CHX-DTPA-scFv78-Fc Targeting Endosialin/Tumor Endothelial Marker 1 (TEM1). *Mol Imaging Biol* **22**, 979–991 (2020). https://doi.org/10.1007/s11307-020-01479-8

**RESULTS**

**Quality control of the radiotracers / Stability**

Based on the amount of the precursor which was used for the radiolabeling and assuming we lose about 20% during the labeling, the apparent molar activities (A_m_) were in the range of 8 to 14 GBq/µmol (not decay corrected).

With regard to the detection of the formation of colloids by ITLC, using the first radio-TLC eluent (0.1 M Na-citrate), [^68^Ga]Ga**-**DOTAGA.Glu.(FAPI)_2_, [^68^Ga]Ga**-**DO3A.Glu.(FAPI)_2_, and ^68^Ga-labelled colloid remain immobilized at the starting point, whereas free gallium-68 moves with the mobile phase. When the second eluent (MeOH / 1 M ammonium acetate (1/1, v/v) is used, only the [^68^Ga]Ga**-**DOTAGA.Glu.(FAPI)_2_, [^68^Ga]Ga**-**DO3A.Glu.(FAPI)_2_ move with the mobile phase / solvent front. The same systems have been applied for the ^177^Lu-labelled tracers.


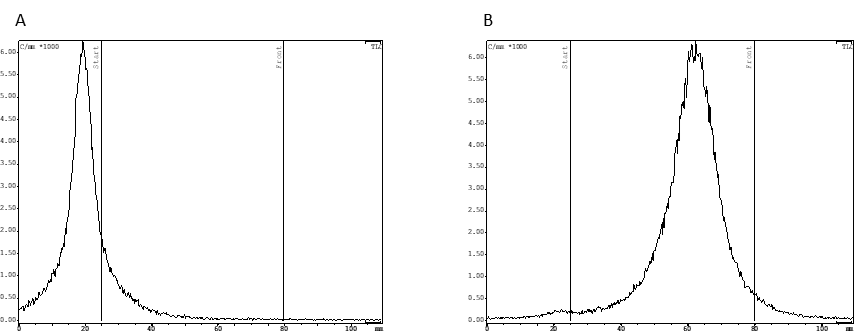


**Figure S1.** Representative TLC profiles in (A) Sodium Citrate and (B) Ammonium Acetate:MeOH of [^68^Ga]Ga- DOTAGA.Glu.(FAPI)_2._


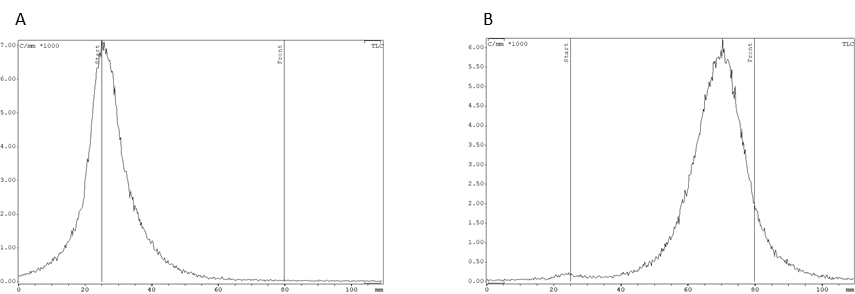


**Figure S2**. Representative TLC profiles in (A) Sodium Citrate and (B) Ammonium Acetate:MeOH of [^68^Ga]Ga- DO3A.Glu.(FAPI)_2._

_
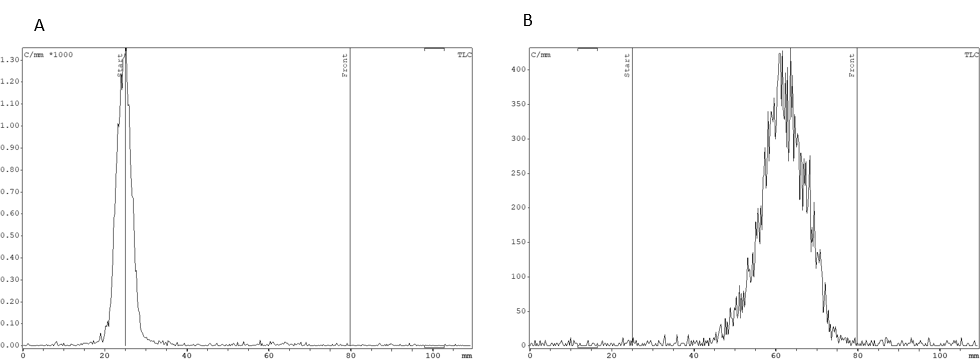
_

**Figure S3**. Representative TLC profiles in (A) Sodium Citrate and (B) Ammonium Acetate:MeOH of [^177^Lu]Lu- DOTAGA.Glu.(FAPI)_2._


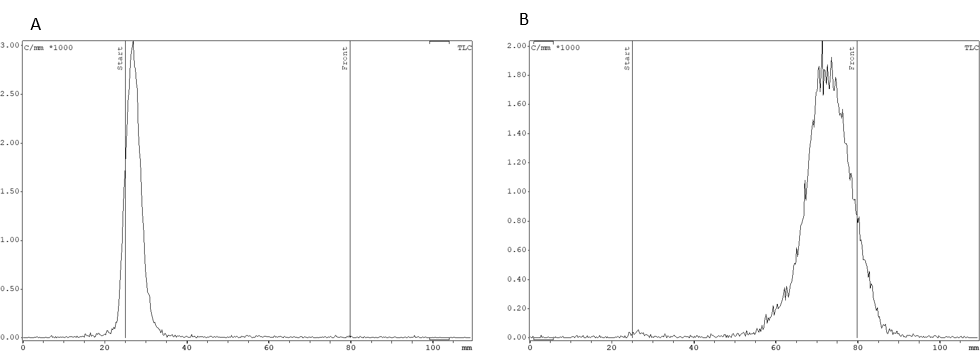


**Figure S4**. Representative TLC profiles in (A) Sodium Citrate and (B) Ammonium Acetate:MeOH of [^177^Lu]Lu- DO3A.Glu.(FAPI)_2._

**Figure S5**. Representative radio-HPLC chromatograms of [^68^Ga]Ga**-**DOTAGA.Glu.(FAPI)_2_, [^68^Ga]Ga**-**DO3A.Glu.(FAPI)_2_**
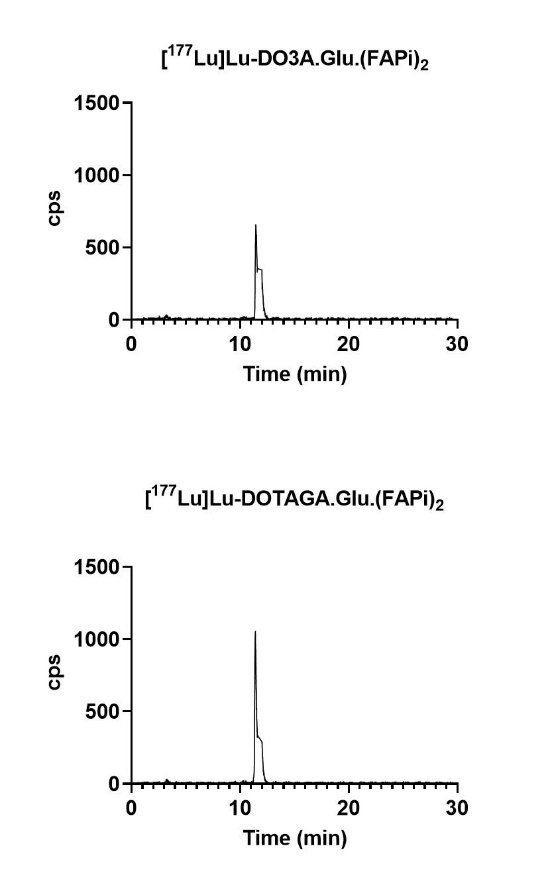
**
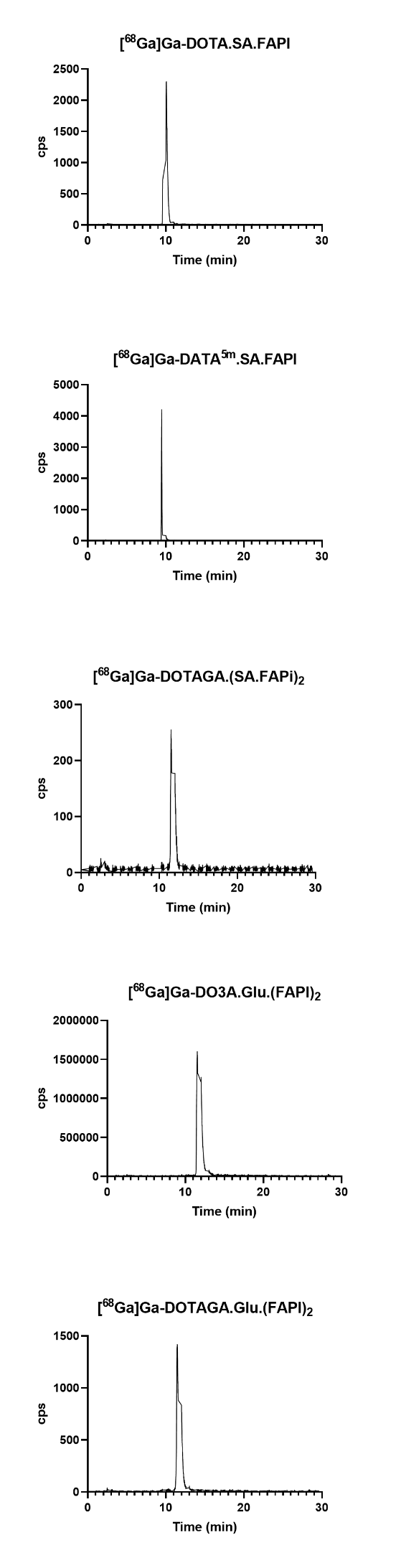
_,_ [^177^Lu]Lu-DOTAGA.Glu.(FAPI)_2_ and [^177^Lu]Lu-DO3A.Glu.(FAPI)_2._

**Biodistribution Studies**

**Table S1**. Biodistribution data of [^68^Ga]Ga-DOTAGA.Glu.(FAPI)_2_ and [^68^Ga]Ga-DO3A.Glu.(FAPI)_2_ in PC3 xenografts at 1, 2, and 3 h p.i along with blocking studies data at 1 h p.i. Data have been calculated as %I.A./g of tissue and are presented as mean ± SD (n = 3).

| **[^68^Ga]Ga-DOTAGA.Glu.(FAPI)_2_** | | | | | **[^68^Ga]Ga-DO3A.Glu.(FAPI)_2_** | | | | |
| --- | --- | --- | --- | --- | --- | --- | --- | --- | --- |
| Organs | **1h** | **2h** | **3h** | **Blocking 1h** | | **1h** | **2h** | **3h** | **Blocking 1h** |
| **Blood**  **Heart**  **Gallbladder**  **Liver**  **Spleen**  **Lung**  **Kidney**  **Stomach**  **Intestine**  **Adrenal**  **Pancreas**  **Muscle**  **Bone**  **Tumor**  **Salivary glands** | 3.5±0.2  1.6±0.2  3.2±0.6  3±0.4  1.6±0.01  3.7±0.6  8.3±0.7  1.3±0.2  1.4±0.2  3.6±0.6  2.5±0.2  1.9±0.2  4.9±0.8  18.2±3.8  8.2±0.5 | 2.6±0.1  1±0.01  2.3±0.4  1.4±0.04  1±0.1  1.7±0.2  4.8±0.5  0.9±0.04  1±0.03  4.2±0.2  2.3±0.1  1.7±0.3  6.4±1.2  17.8±0.5  6.5±1.1 | 2.5±0.1  1±0.2  2.6±1.1  1.6±0.2  1.1±0.2  1.4±0.1  4.4±0.4  1±0.1  1.1±0.1  3.9±2  2±0.1  1.6±0.2  6.7±0.1  18.4±2.1  6±0.1 | 1.7±0.5  0.6±0.1  5.5±0.6  0.6±0.2  0.5±0.1  1.3±0.5  5.6±1.8  0.5±0.1  0.6±0.3  0.7±0.1  0.3±0.1  0.2±0.1  0.3±0.2  1.4±0.5  0.7±0.1 | | 2±0.1  1±0.1  2.3±1.8  4.2±1.2  1.3±0.3  3.8±1  6.7±1.7  0.9±0.1  0.9±0.2  3.6±1.1  1.8±0.1  2±0.03  5±3.1  19±2.4  6.4±0.5 | 2.2±0.1  0.8±0.03  3.5±1.8  3.3±0.9  1.6±0.4  2.5±0.6  4.4±0.8  0.9±0.1  1±0.2  2.5±0.8  1.7±0.1  1.3±0.3  6.3±1  19.6±0.7  5.2±0.1 | 2.2±0.1  0.7±0.1  1.7±0.9  2.8±0.3  1.4±0.3  1.7±0.3  3.4±0.4  0.7±0.02  0.9±0.2  2.4±0.8  1.4±0.1  1.1±0.1  5.8±1  17.5±1.1  5±1.4 | 2.3±0.5  0.7±0.2  3.5±0.3  3.2±0.7  1±0.1  2.1±0.4  6.2±1.2  0.8±0.3  0.8±0.3  0.1±0.6  0.4±0.1  0.3±0.01  0.3±0.1  1.3±0.2  0.7±0.1 |

| **Tumor/Blood** | 5.2±1.1 | 6.8±0.3 | 7.5±0.7 |  | 9.7±1.2 | 8.9±0.7 | 8.2±0.8 |  |
| --- | --- | --- | --- | --- | --- | --- | --- | --- |
| **Tumor/Liver** | 6.3±2 | 12.8±0.7 | 11.5±1.7 |  | 4.8±1.1 | 6.2±1.8 | 6.4±0.5 |  |
| **Tumor/Kidney** | 2.2±0.6 | 3.7±0.4 | 4.3±0.8 |  | 2.9±0.6 | 4.6±1 | 5.2±0.4 |  |
| **Tumor/Bone**  **Tumor/Salivary glands**  **B** | 3.9±1.5  2.2±0.4 | 2.9±0.6  2.8±0.4 | 2.6±0.2  3.1±0.3 |  | 4.8±2.5  3±0.6 | 3.2±0.5  3.8±0.2 | 3±0.3  3.8±1.4 |  |
|  |  |  |  |  |  |  |  |  |

**Table S2**. Biodistribution data and tumor to organ ratios of [^177^Lu]Lu-DOTAGA.Glu.(FAPI)_2_ in PC3 xenografts at 4. 24. 48. 72 and 96 h p.i along with blocking studies data at 4 h p.i. Data have been calculated as %I.A./g of tissue and are presented as mean ± SD (n = 3).

| **[^177^Lu]Lu-DOTAGA.Glu.(FAPI)_2_** | | | | | | | |
| --- | --- | --- | --- | --- | --- | --- | --- |
|  | **4h** | **24h** | **48h** | **72h** | **96h** | **Blocking 4h** |  |
| **Blood**  **Heart**  **Gallbladder**  **Liver**  **Spleen**  **Lung**  **Kidney**  **Stomach**  **Intestine**  **Adrenal**  **Pancreas**  **Muscle**  **Bone**  **Tumor**  **Salivary glands** | 2.1±0.1  0.9±0.1  2.7±1.6  1.7±0.2  0.9±0.1  1.1±0.1  2.6±0.4  0.8±0.1  0.8±0.3  3±0.2  1.9±0.1  1.4±0.3  4±0.3  16.2±2.5  6.2±1.2 | 0.9±0.2  0.6±0.1  0.4±0.1  2.1±0.2  0.7±0.2  0.6±0.2  1.3±0.2  0.4±0.1  0.4±0.1  1.9±0.2  0.9±0.7  0.8±0.1  2.5±0.1  7.3±0.6  2.9±0.5 | 0.2±0.03  0.4±0.04  0.1±0.1  1.9±0.5  0.5±0.04  0.3±0.1  1±0.2  0.3±0.03  0.2±0.01  1.3±0.3  0.6±0.1  0.5±0.1  1.3±0.1  5.1±0.1  2.2±0.3 | 0.1±0.03  0.3±0.1  0.1±0.1  1.4±0.4  0.3±0.1  0.1±0.02  0.6±0.1  0.1±0.02  0.1±0.02  0.9±0.02  0.4±0.03  0.2±0.02  0.9±0.2  3.2±0.3  0.7±0.04 | 0.03±0  0.2±0.02  0.1±0.02  1.3±0.1  0.3±0.03  0.1±0.02  0.4±0.04  0.1±0.01  0.1±0.02  0.5±0.01  0.2±0.02  0.1±0.1  0.6±0.1  2.2±0.2  0.7±0.1 | 0.03±0.01  0.2±0.04  0.2±0.2  1.2±0.5  0.3±0.1  0.1±0.04  0.5±0.1  0.09±0.02  0.1±0.02  0.8±0.5  0.2±0.2  0.2±0.04  0.7±0.1  2.9±1.8  0.6±0.2 |  |
|  |  |  |  |  |  |  |  |
| **Tumor/Blood**  **Tumor/Liver**  **Tumor/Kidney**  **Tumor/Bone**  **Tumor/Salivary glands** | 8±1.6  9.4±0.9  6.4±0.9  4.1±0.4  2.7±0.9 | 8.7±1.1  3.5±0.2  5.6±0.7  2.9±0.3  2.6±0.5 | 24.5±2.3  2.9±0.7  5.4±1  3.9±0.3  2.4±0.3 | 38.4±8.4  2.4±1532  5.6±0.3  3.5±0.6  4.5±0.7 | 72±5.5  1.6±0.1  5.5±0.4  3.6±0.3  3.3±0.1 |  |  |

**Table S3**. Biodistribution data and tumor to organ ratios of [^177^Lu]Lu-DO3A.Glu.(FAPI)_2_ in PC3 xenografts at 4. 24. 48. 72 and 96 h p.i along with blocking studies data at 4 h p.i. Data have been calculated as %I.A./g of tissue and are presented as mean ± SD (n = 3).

| **[^177^Lu]Lu-DO3A.Glu.(FAPI)_2_** | | | | | | | | | | | | | |
| --- | --- | --- | --- | --- | --- | --- | --- | --- | --- | --- | --- | --- | --- |
|  | **4h** | | **24h** | | **48h** | | **72h** | | **96h** | | **Blocking 4h** | |  |
| **Blood**  **Heart**  **Gallbladder**  **Liver**  **Spleen**  **Lung**  **Kidney**  **Stomach**  **Intestine**  **Adrenal**  **Pancreas**  **Muscle**  **Bone**  **Tumor**  **Salivary glands** | | 2.3±0.3  1.2±0.5  nd  1.7±0.1  1.2±0.1  1.6±0.02  1.5±0.4  0.9±0.2  0.9±0.4  3.7±1  2.3±0.4  1.9±0.3  5.2±0.2  15±1.3  8.2±1.3 | | 0.9±0.1  0.4±0.03  1.3±0.03  1.7±0.2  0.8±0.05  0.6±0.03  0.9±0.1  0.4±0.03  0.4±0.1  1.5±0.2  1±0.05  1±0.1  2±0.1  7.5±1.2  3.9±0.3 | | 0.2±0.03  0.1±0.01  0.6±0.1  1.1±0.2  0.5±0.05  0.2±0.1  0.4±0.1  0.2±0.05  0.1±0.04  1.1±0.5  0.3±0.01  0.4±0.04  1±0.1  3.1±0.5  1.1±0.3 | | 0.06±0  0.04±0.01  0.4±0.2  0.8±0.1  0.3±0.04  0.1±0.01  0.2±0.02  0.05±0  0.05±0.01  0.3±0.1  0.1±0.02  0.1±0.01  0.4±0.005  1.4±0.1  0.3±0.1 | | 0.02±0.01  0.02±0.01  0.1±0.02  0.7±0.1  0.4±0.05  0.1±0.1  0.1±0.01  0.03±0.01  0.02±0.01  0.2±0.1  0.03±0.01  0.04±0  0.3±0.1  1±0.03  0.1±0.03 | | 0.2±0.04  0.1±0.01  nd  1.1±0.01  0.4±0.03  0.5±0.1  2.8±0.2  0.2±0.1  0.4±0.02  0.3±0.04  0.1±0.01  0.1±0.01  0.4±0.1  1.1±0.4  0.3±0.01 | |
| **Tumor/Blood**  **Tumor/Liver**  **Tumor/Kidney**  **Tumor/Bone**  **Tumor/Salivary glands** | | 6.6±1.4  9±1.4  10.3±2.7  2.9±0.2  1.9±0.4 | | 8.2±0.9  4.4±0.3  7.9±0.7  3.7±0.7  1.9±0.3 | | 13.3±1.3  2.9±0.1  7.6±1.4  3.2±0.5  2.9±0.8 | | 24.4±3  1.9±0.3  8.2±0.8  3.9±0.7  5±1.8 | | 51.5±0.7  1.4±0.1  7.9±0.6  3.7±0.7  7.4±1.4 | |  | |

**Organs clearance**

**
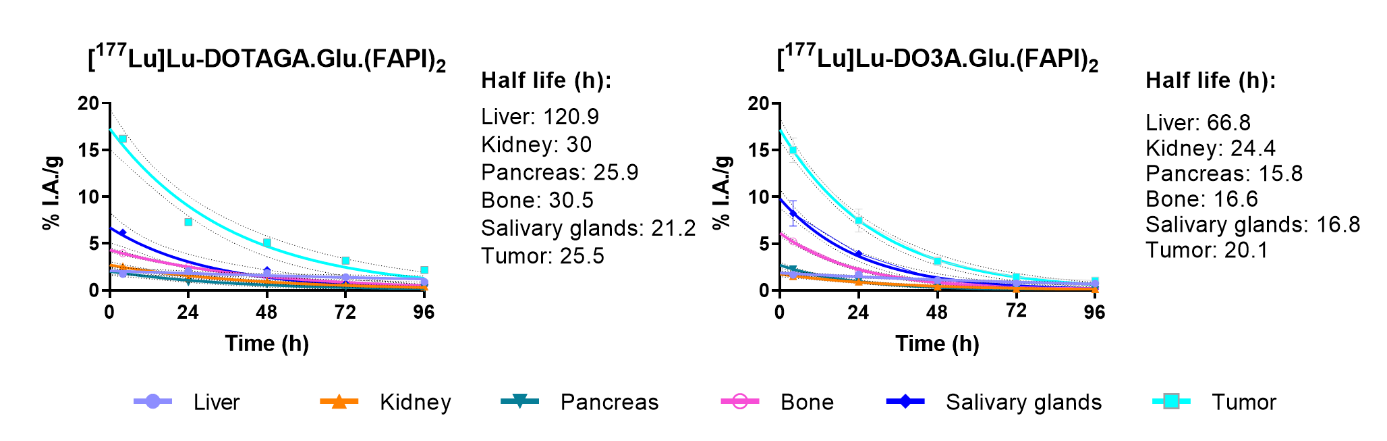
**

**Figure S7.** Crucial organ clearance kinetics of [^177^Lu]Lu-DOTAGA.Glu.(FAPI)_2_ and [^177^Lu]Lu-DO3A.Glu.(FAPI)_2_ at 4, 24, 48, 72 and 96 hours p.i. along with half-lives for each organ.

**Table S4.** The 95% confidence intervals for organs clearance (half-lives in hours) of [^177^Lu]Lu-DOTAGA.Glu.(FAPI)_2_ and [^177^Lu]Lu-DO3A.Glu.(FAPI)_2_.

| **[^177^Lu]Lu-DOTAGA.Glu.(FAPI)_2_** | | **[^177^Lu]Lu-DO3A.Glu.(FAPI)_2_** |
| --- | --- | --- |
| **Liver** | 70.61 to 362.5 | 51.46 to 92.13 |
| **Kidney** | 23.41 to 38.84 | 18.73 to 31.89 |
| **Pancreas** | 16.87 to 39.95 | 12.66 to 19.50 |
| **Bone** | 27.60 to 33.77 | 14.85 to 18.53 |
| **Salivary glands** | 12.06 to 34.90 | 13.95 to 20.7 |
| **Tumor** | 19.85 to 32.80 | 17.58 to 22.85 |

**Dosimetry**

**Table S5.** TIAC for [^177^Lu]Lu-DOTAGA.Glu.(FAPI)_2_

|  | TIAC average | | TIAC lower | | TIAC upper | |
| --- | --- | --- | --- | --- | --- | --- |
| Organ | M | M | F | F | F | F |
| heart content | 0.0945 | 0.0834 | 0.0902 | 0.0796 | 0.0991 | 0.0875 |
| heart wall | 0.0965 | 0.0889 | 0.0962 | 0.0887 | 0.1006 | 0.0927 |
| liver | 3.3017 | 3.1244 | 3.0686 | 2.9038 | 3.6270 | 3.4322 |
| spleen | 0.0456 | 0.0480 | 0.0352 | 0.0371 | 0.0590 | 0.0622 |
| lung | 0.4829 | 0.4651 | 0.4408 | 0.4246 | 0.5240 | 0.5047 |
| kidney | 0.1339 | 0.1445 | 0.1287 | 0.1389 | 0.1397 | 0.1508 |
| stomach | 0.0557 | 0.0626 | 0.0538 | 0.0605 | 0.0576 | 0.0648 |
| Small Intestine | 0.1684 | 0.1803 | 0.1381 | 0.1478 | 0.2055 | 0.2200 |
| Right column | 0.1129 | 0.1396 | 0.0925 | 0.1144 | 0.1377 | 0.1703 |
| Left Column | 0.1129 | 0.1373 | 0.0925 | 0.1125 | 0.1377 | 0.1675 |
| Rectum | 0.1129 | 0.1420 | 0.0925 | 0.1164 | 0.1377 | 0.1733 |
| adrenal | 0.0009 | 0.0011 | 0.0009 | 0.0010 | 0.0010 | 0.0011 |
| pancreas | 0.0441 | 0.0460 | 0.0419 | 0.0437 | 0.0462 | 0.0482 |
| bone | 5.5459 | 4.9073 | 5.2161 | 4.6154 | 5.8733 | 5.1970 |
| Red marrow | 0.2169 | 0.2030 | 0.2068 | 0.1936 | 0.2274 | 0.2128 |
| salivary glands | 0.1010 | 0.1012 | 0.0960 | 0.0962 | 0.1020 | 0.1022 |
| ROB | 15.0586 | 18.6076 | 12.9983 | 13.7147 | 17.0071 | 17.7951 |
| Total Frac IA | 25.6850 | 25.6850 | 22.8890 | 22.8890 | 28.4823 | 28.4823 |

**Table S6.** TIAC for [^177^Lu]Lu-DO3A.Glu.(FAPI)_2_

|  | TIAC average | | TIAC lower | | TIAC upper | |
| --- | --- | --- | --- | --- | --- | --- |
| Organ | M | M | F | F | F | F |
| heart content | 0.1028 | 0.0908 | 0.1002 | 0.0884 | 0.1054 | 0.0930 |
| heart wall | 0.0357 | 0.0329 | 0.0339 | 0.0312 | 0.0376 | 0.0346 |
| liver | 1.1865 | 1.1228 | 1.1343 | 1.0733 | 1.2328 | 1.1666 |
| spleen | 0.0881 | 0.0929 | 0.0838 | 0.0884 | 0.0915 | 0.0965 |
| lung | 0.4477 | 0.4313 | 0.3844 | 0.3702 | 0.5107 | 0.4919 |
| kidney | 0.0664 | 0.0717 | 0.0640 | 0.0690 | 0.0689 | 0.0743 |
| stomach | 0.0446 | 0.0501 | 0.0442 | 0.0498 | 0.0463 | 0.0521 |
| Small Intestine | 0.0958 | 0.1025 | 0.0880 | 0.0942 | 0.1050 | 0.1124 |
| Right column | 0.0642 | 0.0794 | 0.0588 | 0.0727 | 0.0703 | 0.0870 |
| Left Column | 0.0642 | 0.0781 | 0.0588 | 0.0715 | 0.0703 | 0.0855 |
| Rectum | 0.0642 | 0.0808 | 0.0588 | 0.0740 | 0.0703 | 0.0885 |
| adrenal | 0.0007 | 0.0008 | 0.0007 | 0.0008 | 0.0008 | 0.0009 |
| pancreas | 0.0300 | 0.0313 | 0.0290 | 0.0303 | 0.0310 | 0.0323 |
| bone | 3.1852 | 2.8185 | 3.0873 | 2.7318 | 3.2849 | 2.9067 |
| Red marrow | 0.2359 | 0.2208 | 0.2298 | 0.2150 | 0.2417 | 0.2262 |
| salivary glands | 0.0706 | 0.0707 | 0.0634 | 0.0635 | 0.0773 | 0.0774 |
| ROB | 15.5056 | 16.8035 | 14.7254 | 15.1203 | 16.1340 | 16.5527 |
| Total Frac IA | 21.2881 | 21.2881 | 20.2445 | 20.2445 | 22.1786 | 22.1786 |

**Table S7.** Target organ absorbed dose for [^177^Lu]Lu-DOTAGA.Glu.(FAPI)_2_ for ICRP 89 Adult Male

|  |  |  |  |
| --- | --- | --- | --- |
| Target Organ | AD average | AD lower | AD upper |
| Adrenals | 1.17E-02 | 1.11E-02 | 1.29E-02 |
| Brain | 1.98E-02 | 1.71E-02 | 2.23E-02 |
| Esophagus | 2.11E-02 | 1.84E-02 | 2.37E-02 |
| Eyes | 1.97E-02 | 1.71E-02 | 2.22E-02 |
| Gallbladder Wall | 2.49E-02 | 2.19E-02 | 2.80E-02 |
| Left colon | 8.56E-02 | 7.11E-02 | 1.02E-01 |
| Small Intestine | 4.17E-02 | 3.51E-02 | 4.89E-02 |
| Stomach Wall | 3.05E-02 | 3.51E-02 | 3.35E-02 |
| Right colon | 5.39E-02 | 2.75E-02 | 6.38E-02 |
| Rectum | 8.54E-02 | 4.52E-02 | 1.02E-01 |
| Heart Wall | 3.66E-02 | 3.58E-02 | 3.84E-02 |
| Kidneys | 4.08E-02 | 3.90E-02 | 4.28E-02 |
| Liver | 1.64E-01 | 1.53E-01 | 1.81E-01 |
| Lungs | 3.74E-02 | 3.41E-02 | 4.06E-02 |
| Pancreas | 3.11E-02 | 2.94E-02 | 3.29E-02 |
| Prostate | 2.04E-02 | 1.77E-02 | 2.31E-02 |
| Salivary Glands | 1.03E-01 | 9.80E-02 | 1.05E-01 |
| Red Marrow | 2.38E-02 | 2.15E-02 | 2.61E-02 |
| Osteogenic Cells | 9.26E-02 | 8.52E-02 | 9.97E-02 |
| Spleen | 2.87E-02 | 2.24E-02 | 3.67E-02 |
| Testes | 1.95E-02 | 1.69E-02 | 2.20E-02 |
| Thymus | 2.03E-02 | 1.76E-02 | 2.29E-02 |
| Thyroid | 2.02E-02 | 1.75E-02 | 2.27E-02 |
| Urinary Bladder Wall | 2.03E-02 | 1.75E-02 | 2.29E-02 |
| Total Body | 3.25E-02 | 2.90E-02 | 3.61E-02 |
| Effective Dose | 3.40E-02 | 3.02E-02 | 3.81E-02 |

**Table S8.** Target organ absorbed dose for [^177^Lu]Lu-DOTAGA.Glu.(FAPI)_2_ for ICRP 89 Adult Female

|  |  |  |  |
| --- | --- | --- | --- |
| Target Organ | AD average | AD lower | AD upper |
| Adrenals | 1.46E-02 | 1.29E-02 | 1.51E-02 |
| Brain | 2.94E-02 | 2.18E-02 | 2.82E-02 |
| Breasts | 2.88E-02 | 2.13E-02 | 2.76E-02 |
| Esophagus | 3.06E-02 | 2.30E-02 | 2.96E-02 |
| Eyes | 2.94E-02 | 2.18E-02 | 2.82E-02 |
| Gallbladder Wall | 3.30E-02 | 2.48E-02 | 3.21E-02 |
| Left colon | 1.05E-01 | 2.29E-02 | 1.20E-01 |
| Small Intestine | 5.84E-02 | 2.27E-02 | 6.34E-02 |
| Stomach Wall | 4.24E-02 | 2.30E-02 | 4.18E-02 |
| Right colon | 6.88E-02 | 2.31E-02 | 7.60E-02 |
| Rectum | 1.07E-01 | 2.26E-02 | 1.23E-01 |
| Heart Wall | 4.42E-02 | 4.30E-02 | 4.60E-02 |
| Kidneys | 4.97E-02 | 4.71E-02 | 5.18E-02 |
| Liver | 2.00E-01 | 1.85E-01 | 2.19E-01 |
| Lungs | 4.59E-02 | 4.15E-02 | 4.95E-02 |
| Ovaries | 3.09E-02 | 2.26E-02 | 2.97E-02 |
| Pancreas | 3.90E-02 | 3.63E-02 | 4.09E-02 |
| Salivary Glands | 1.26E-01 | 1.19E-01 | 1.27E-01 |
| Red Marrow | 3.24E-02 | 2.65E-02 | 3.22E-02 |
| Osteogenic Cells | 8.92E-02 | 7.82E-02 | 9.16E-02 |
| Spleen | 3.53E-02 | 2.73E-02 | 4.47E-02 |
| Thymus | 3.04E-02 | 2.27E-02 | 2.92E-02 |
| Thyroid | 2.95E-02 | 2.19E-02 | 2.83E-02 |
| Urinary Bladder Wall | 3.05E-02 | 2.23E-02 | 2.93E-02 |
| Uterus | 3.10E-02 | 2.25E-02 | 2.99E-02 |
| Total Body | 4.42E-02 | 3.47E-02 | 4.42E-02 |
| Effective dose | 4.71E-02 | 3.17E-02 | 4.95E-02 |

**Table S9.** Target organ absorbed dose for [^177^Lu]Lu-DO3A.Glu.(FAPI)_2_ for ICRP 89 Adult Male

|  |  |  |  |
| --- | --- | --- | --- |
| Target Organ | AD average | AD lower | AD upper |
| Adrenals | 7.77E-03 | 7.60E-03 | 8.52E-03 |
| Brain | 2.00E-02 | 1.90E-02 | 2.08E-02 |
| Esophagus | 2.06E-02 | 1.96E-02 | 2.15E-02 |
| Eyes | 2.00E-02 | 1.90E-02 | 2.08E-02 |
| Gallbladder Wall | 2.20E-02 | 2.09E-02 | 2.29E-02 |
| Left colon | 5.77E-02 | 5.35E-02 | 6.20E-02 |
| Small Intestine | 3.27E-02 | 3.07E-02 | 3.47E-02 |
| Stomach Wall | 2.83E-02 | 2.72E-02 | 2.95E-02 |
| Right colon | 3.94E-02 | 3.68E-02 | 4.20E-02 |
| Rectum | 5.76E-02 | 5.35E-02 | 6.20E-02 |
| Heart Wall | 2.04E-02 | 1.96E-02 | 2.12E-02 |
| Kidneys | 2.09E-02 | 2.01E-02 | 2.17E-02 |
| Liver | 6.00E-02 | 5.73E-02 | 6.23E-02 |
| Lungs | 3.40E-02 | 2.94E-02 | 3.86E-02 |
| Pancreas | 2.12E-02 | 2.05E-02 | 2.20E-02 |
| Prostate | 2.07E-02 | 1.97E-02 | 2.16E-02 |
| Salivary Glands | 7.27E-02 | 6.54E-02 | 7.95E-02 |
| Red Marrow | 2.43E-02 | 2.33E-02 | 2.51E-02 |
| Osteogenic Cells | 6.64E-02 | 6.38E-02 | 6.86E-02 |
| Spleen | 5.27E-02 | 5.01E-02 | 5.47E-02 |
| Testes | 2.00E-02 | 1.90E-02 | 2.08E-02 |
| Thymus | 2.03E-02 | 1.93E-02 | 2.12E-02 |
| Thyroid | 2.04E-02 | 1.94E-02 | 2.13E-02 |
| Urinary Bladder Wall | 2.07E-02 | 1.96E-02 | 2.15E-02 |
| Total Body | 2.71E-02 | 2.57E-02 | 2.82E-02 |
| Effective Dose | 2.56E-02 | 2.39E-02 | 2.72E-02 |

**Table S10.** Target organ absorbed dose for [^177^Lu]Lu-DO3A.Glu.(FAPI)_2_ for ICRP 89 Adult Female

|  |  |  |  |
| --- | --- | --- | --- |
| Target Organ | AD average | AD lower | AD upper |
| Adrenals | 9.96E-03 | 9.65E-03 | 1.07E-02 |
| Brain | 2.63E-02 | 2.37E-02 | 2.60E-02 |
| Breasts | 2.57E-02 | 2.32E-02 | 2.54E-02 |
| Esophagus | 2.66E-02 | 2.40E-02 | 2.63E-02 |
| Eyes | 2.63E-02 | 2.37E-02 | 2.60E-02 |
| Gallbladder Wall | 2.80E-02 | 2.53E-02 | 2.77E-02 |
| Left colon | 6.93E-02 | 6.31E-02 | 7.29E-02 |
| Small Intestine | 4.29E-02 | 3.90E-02 | 4.41E-02 |
| Stomach Wall | 3.65E-02 | 3.38E-02 | 3.66E-02 |
| Right colon | 4.88E-02 | 4.43E-02 | 5.05E-02 |
| Rectum | 7.09E-02 | 6.45E-02 | 7.47E-02 |
| Heart Wall | 2.49E-02 | 2.37E-02 | 2.58E-02 |
| Kidneys | 2.54E-02 | 2.44E-02 | 2.63E-02 |
| Liver | 7.30E-02 | 6.97E-02 | 7.57E-02 |
| Lungs | 4.16E-02 | 3.59E-02 | 4.71E-02 |
| Ovaries | 2.76E-02 | 2.48E-02 | 2.72E-02 |
| Pancreas | 2.62E-02 | 2.52E-02 | 2.70E-02 |
| Salivary Glands | 8.81E-02 | 7.92E-02 | 9.63E-02 |
| Red Marrow | 3.06E-02 | 2.85E-02 | 3.07E-02 |
| Osteogenic Cells | 6.24E-02 | 5.88E-02 | 6.32E-02 |
| Spleen | 6.42E-02 | 6.10E-02 | 6.66E-02 |
| Thymus | 2.70E-02 | 2.43E-02 | 2.67E-02 |
| Thyroid | 2.64E-02 | 2.37E-02 | 2.60E-02 |
| Urinary Bladder Wall | 2.73E-02 | 2.46E-02 | 2.69E-02 |
| Uterus | 2.76E-02 | 2.48E-02 | 2.72E-02 |
| Total Body | 3.44E-02 | 3.14E-02 | 3.44E-02 |
| Effective dose | 3.49E-02 | 3.18E-02 | 3.61E-02 |

**Table S11.** Target organ absorbed dose for [^161^Tb]Tb-DOTAGA.Glu.(FAPI)_2_ for ICRP 89 Adult Male

|  |  |  |  |
| --- | --- | --- | --- |
| Target Organ | AD average | AD lower | AD upper |
| Adrenals | 1.63E-02 | 1.54E-02 | 1.78E-02 |
| Brain | 2.76E-02 | 2.38E-02 | 3.10E-02 |
| Esophagus | 2.88E-02 | 2.50E-02 | 3.23E-02 |
| Eyes | 2.76E-02 | 2.38E-02 | 3.10E-02 |
| Gallbladder Wall | 3.50E-02 | 3.06E-02 | 3.90E-02 |
| Left colon | 1.18E-01 | 2.44E-02 | 3.18E-02 |
| Small Intestine | 5.69E-02 | 2.43E-02 | 3.17E-02 |
| Stomach Wall | 4.15E-02 | 2.47E-02 | 3.20E-02 |
| Right colon | 7.38E-02 | 2.50E-02 | 3.24E-02 |
| Rectum | 1.17E-01 | 2.40E-02 | 3.13E-02 |
| Heart Wall | 5.00E-02 | 4.90E-02 | 5.23E-02 |
| Kidneys | 5.60E-02 | 5.34E-02 | 5.85E-02 |
| Liver | 2.29E-01 | 2.13E-01 | 2.51E-01 |
| Lungs | 5.19E-02 | 4.73E-02 | 5.64E-02 |
| Pancreas | 4.25E-02 | 3.98E-02 | 4.44E-02 |
| Prostate | 2.77E-02 | 2.36E-02 | 3.09E-02 |
| Salivary Glands | 1.43E-01 | 1.35E-01 | 1.44E-01 |
| Red Marrow | 3.37E-02 | 3.03E-02 | 3.68E-02 |
| Osteogenic Cells | 1.24E-01 | 1.14E-01 | 1.33E-01 |
| Spleen | 3.92E-02 | 3.05E-02 | 5.01E-02 |
| Testes | 2.65E-02 | 2.29E-02 | 2.99E-02 |
| Thymus | 2.77E-02 | 2.40E-02 | 3.11E-02 |
| Thyroid | 2.76E-02 | 2.39E-02 | 3.11E-02 |
| Urinary Bladder Wall | 2.75E-02 | 2.36E-02 | 3.09E-02 |
| Total Body | 4.51E-02 | 3.94E-02 | 4.88E-02 |
| Effective Dose | 4.69E-02 | 3.29E-02 | 4.01E-02 |

**Table S12.** Target organ absorbed dose for [^161^Tb]Tb-DOTAGA.Glu.(FAPI)_2_ for ICRP 89 Adult Female

|  |  |  |  |
| --- | --- | --- | --- |
| Target Organ | AD average | AD lower | AD upper |
| Adrenals | 2.06E-02 | 1.84E-02 | 2.14E-02 |
| Brain | 4.11E-02 | 3.05E-02 | 3.94E-02 |
| Breasts | 3.94E-02 | 2.92E-02 | 3.78E-02 |
| Esophagus | 4.21E-02 | 3.17E-02 | 4.07E-02 |
| Eyes | 4.11E-02 | 3.05E-02 | 3.94E-02 |
| Gallbladder Wall | 4.47E-02 | 3.38E-02 | 4.34E-02 |
| Left colon | 4.20E-02 | 3.13E-02 | 4.04E-02 |
| Small Intestine | 4.17E-02 | 3.09E-02 | 4.00E-02 |
| Stomach Wall | 4.21E-02 | 3.14E-02 | 4.05E-02 |
| Right colon | 4.24E-02 | 3.15E-02 | 4.07E-02 |
| Rectum | 4.17E-02 | 3.08E-02 | 3.99E-02 |
| Heart Wall | 6.03E-02 | 5.88E-02 | 6.28E-02 |
| Kidneys | 6.83E-02 | 6.50E-02 | 7.12E-02 |
| Liver | 2.78E-01 | 2.58E-01 | 3.05E-01 |
| Lungs | 6.37E-02 | 5.77E-02 | 6.88E-02 |
| Ovaries | 4.17E-02 | 3.08E-02 | 3.99E-02 |
| Pancreas | 5.34E-02 | 5.00E-02 | 5.59E-02 |
| Salivary Glands | 1.73E-01 | 1.64E-01 | 1.75E-01 |
| Red Marrow | 4.59E-02 | 3.77E-02 | 4.56E-02 |
| Osteogenic Cells | 1.23E-01 | 1.07E-01 | 1.26E-01 |
| Spleen | 4.83E-02 | 3.74E-02 | 6.13E-02 |
| Thymus | 4.16E-02 | 3.11E-02 | 4.01E-02 |
| Thyroid | 4.05E-02 | 3.00E-02 | 3.88E-02 |
| Urinary Bladder Wall | 4.10E-02 | 3.03E-02 | 3.92E-02 |
| Uterus | 4.14E-02 | 3.06E-02 | 3.96E-02 |
| Total Body | 6.01E-02 | 4.83E-02 | 5.98E-02 |
| Effective dose | 5.28E-02 | 4.39E-02 | 5.38E-02 |

**Table S13.** Target organ absorbed dose for [^161^Tb]Tb-DO3A.Glu.(FAPI)_2_ for ICRP 89 Adult Male

|  |  |  |  |
| --- | --- | --- | --- |
| Target Organ | AD average | AD lower | AD upper |
| Adrenals | 1.05E-02 | 1.03E-02 | 1.15E-02 |
| Brain | 2.80E-02 | 2.66E-02 | 2.91E-02 |
| Esophagus | 2.84E-02 | 2.69E-02 | 2.96E-02 |
| Eyes | 2.80E-02 | 2.66E-02 | 2.91E-02 |
| Gallbladder Wall | 3.02E-02 | 2.87E-02 | 3.15E-02 |
| Left colon | 2.85E-02 | 2.71E-02 | 2.97E-02 |
| Small Intestine | 2.85E-02 | 2.71E-02 | 2.97E-02 |
| Stomach Wall | 2.84E-02 | 2.69E-02 | 2.95E-02 |
| Right colon | 2.86E-02 | 2.71E-02 | 2.97E-02 |
| Rectum | 2.84E-02 | 2.70E-02 | 2.96E-02 |
| Heart Wall | 2.78E-02 | 2.67E-02 | 2.89E-02 |
| Kidneys | 2.84E-02 | 2.73E-02 | 2.95E-02 |
| Liver | 8.31E-02 | 7.94E-02 | 8.63E-02 |
| Lungs | 4.73E-02 | 4.08E-02 | 5.36E-02 |
| Pancreas | 2.88E-02 | 2.78E-02 | 2.98E-02 |
| Prostate | 2.81E-02 | 2.67E-02 | 2.92E-02 |
| Salivary Glands | 1.00E-01 | 9.03E-02 | 1.10E-01 |
| Red Marrow | 3.45E-02 | 3.31E-02 | 3.57E-02 |
| Osteogenic Cells | 8.97E-02 | 8.63E-02 | 9.28E-02 |
| Spleen | 7.27E-02 | 6.92E-02 | 7.55E-02 |
| Testes | 2.72E-02 | 2.59E-02 | 2.83E-02 |
| Thymus | 2.79E-02 | 2.64E-02 | 2.90E-02 |
| Thyroid | 2.81E-02 | 2.67E-02 | 2.93E-02 |
| Urinary Bladder Wall | 2.81E-02 | 2.67E-02 | 2.92E-02 |
| Total Body | 3.70E-02 | 3.52E-02 | 3.85E-02 |
| Effective Dose | 2.91E-02 | 2.72E-02 | 3.08E-02 |

**Table S14.** Target organ absorbed dose for [^161^Tb]Tb-DO3A.Glu.(FAPI)_2_ for ICRP 89 Adult Female

|  |  |  |  |
| --- | --- | --- | --- |
| Target Organ | AD average | AD lower | AD upper |
| Adrenals | 1.39E-02 | 1.35E-02 | 1.50E-02 |
| Brain | 3.68E-02 | 3.32E-02 | 3.63E-02 |
| Breasts | 3.54E-02 | 3.19E-02 | 3.49E-02 |
| Esophagus | 3.67E-02 | 3.31E-02 | 3.63E-02 |
| Eyes | 3.68E-02 | 3.32E-02 | 3.63E-02 |
| Gallbladder Wall | 3.79E-02 | 3.42E-02 | 3.74E-02 |
| Left colon | 3.75E-02 | 3.38E-02 | 3.70E-02 |
| Small Intestine | 3.72E-02 | 3.35E-02 | 3.67E-02 |
| Stomach Wall | 3.75E-02 | 3.38E-02 | 3.70E-02 |
| Right colon | 3.76E-02 | 3.39E-02 | 3.71E-02 |
| Rectum | 3.75E-02 | 3.38E-02 | 3.69E-02 |
| Heart Wall | 3.39E-02 | 3.24E-02 | 3.52E-02 |
| Kidneys | 3.48E-02 | 3.33E-02 | 3.60E-02 |
| Liver | 1.01E-01 | 9.66E-02 | 1.05E-01 |
| Lungs | 5.78E-02 | 4.98E-02 | 6.54E-02 |
| Ovaries | 3.75E-02 | 3.37E-02 | 3.69E-02 |
| Pancreas | 3.59E-02 | 3.45E-02 | 3.69E-02 |
| Salivary Glands | 1.22E-01 | 1.09E-01 | 1.33E-01 |
| Red Marrow | 4.37E-02 | 4.07E-02 | 4.38E-02 |
| Osteogenic Cells | 8.70E-02 | 8.18E-02 | 8.81E-02 |
| Spleen | 8.87E-02 | 8.43E-02 | 9.20E-02 |
| Thymus | 3.72E-02 | 3.34E-02 | 3.68E-02 |
| Thyroid | 3.64E-02 | 3.27E-02 | 3.59E-02 |
| Urinary Bladder Wall | 3.70E-02 | 3.33E-02 | 3.64E-02 |
| Uterus | 3.73E-02 | 3.36E-02 | 3.68E-02 |
| Total Body | 4.70E-02 | 4.29E-02 | 4.69E-02 |
| Effective dose | 4.10E-02 | 3.71E-02 | 4.19E-02 |

**Table S15.** IC_50_ values of DOTAGA.Glu.(FAPi)_2_, [^nat^Lu]Lu-DOTAGA.Glu.(FAPi)_2_, DO3A.Glu.(FAPi)_2_, DOTAGA.(SA.FAPi)_2_, [^nat^Lu]Lu-DOTAGA.(SA.FAPi)_2_ and the initial FAP inhibitor UAMC-1110 from previous works (1).

| **Compound** | **DOTAGA.Glu.(FAPi)_2_** | **[^nat^Lu]Lu-DOTAGA.Glu.(FAPi)_2_** | **DO3A.Glu.(FAPi)_2_** | **DOTAGA.(SA.FAPi)_2_** | **[^nat^Lu]Lu-DOTAGA.(SA.FAPi)_2_** | **UAMC-1110** |
| --- | --- | --- | --- | --- | --- | --- |
| *IC_50_(FAP)*/nM | 0.26±0.04 | 0.33±0.02 | 0.60±0.04 | 0.92±0.06 | 1.54±0.15 | 0.43±0.02 |
| *IC_50_(PREP)*/µM | 0.59±0.10 | 0.43±0.16 | 1.00±0.14 | 0.39±0.02 | 0.56±0.04 | 1.80±0.01 |
| *IC_50_(DPP4)/*µM | 1.19±0.08 | 0.65±0.04 | 0.54±0.06 | 0.40±0.07 | 0.63±0.07 | >10 |
| *IC_50_(DPP8)/*µM | 0.029±0.004 | 0.22±0.02 | 1.03±0.18 | 0.42±0.04 | 0.41±0.03 | >10 |
| *IC_50_(DPP9)/*µM | 0.083±0.0015 | 0.19±0.01 | 0.95±0.11 | 0.16±0.02 | 0.18±0.02 | 4.70±0.40 |

1. Martin M, Ballal S, Yadav MP, Bal C, Van Rymenant Y, De Loose J*, et al.* Novel Generation of FAP Inhibitor-Based Homodimers for Improved Application in Radiotheranostics. Cancers (Basel) **2023**;15

**Table S16.** LogD_octanol/PBS-pH7.4_ and Percentage of Human Serum Protein Bound Activity.

|  | **LogD value** | **% of protein binding after 30 minutes** |
| --- | --- | --- |
| **[^68^Ga]Ga-DOTAGA.(SA.FAPI)_2_** | -1.8 ± 0.02 | 18 ± 1.1 |
| **[^177^Lu]Lu-DOTAGA.(SA.FAPI)_2_** | -1.7 ± 0.03 | 25.3 ± 0.8 |
| **[^68^Ga]Ga-DOTAGA.Glu.(FAPI)_2_**  **[^177^Lu]Lu-DOTAGA.Glu.(FAPI)_2_**  **[^68^Ga]Ga-DO3A.Glu.(FAPI)_2_**  **[^177^Lu]Lu-DO3A.Glu.(FAPI)_2_** | -2.9 ± 0.1  -3.0 ± 0.1  -2.2 ± 0.04  -1.7 ± 0.01 | 9.4 ± 2.2  9.2 ± 0.03  9.6 ± 0.2  27.7 ± 0.9 |
